# Mitochondria: a new intervention target for tumor invasion and metastasis

**DOI:** 10.1186/s10020-024-00899-4

**Published:** 2024-08-23

**Authors:** Quanling Zhou, Tingping Cao, Fujun Li, Ming Zhang, Xiaohui Li, Hailong Zhao, Ya Zhou

**Affiliations:** 1 Department of Pathophysiology, Zunyi Medical University, Zunyi Guizhou, 563000 China; 2 Department of Physics, Zunyi Medical University, Zunyi Guizhou, 563000 China; 3 Key Laboratory of Gene Detection and Therapy of Guizhou Province, Zunyi Guizhou, 563000 China

**Keywords:** Mitochondria, Energy metabolism, Tumor, Invasion and metastasis, Signal transduction

## Abstract

Mitochondria, responsible for cellular energy synthesis and signal transduction, intricately regulate diverse metabolic processes, mediating fundamental biological phenomena such as cell growth, aging, and apoptosis. Tumor invasion and metastasis, key characteristics of malignancies, significantly impact patient prognosis. Tumor cells frequently exhibit metabolic abnormalities in mitochondria, including alterations in metabolic dynamics and changes in the expression of relevant metabolic genes and associated signal transduction pathways. Recent investigations unveil further insights into mitochondrial metabolic abnormalities, revealing their active involvement in tumor cell proliferation, resistance to chemotherapy, and a crucial role in tumor cell invasion and metastasis. This paper comprehensively outlines the latest research advancements in mitochondrial structure and metabolic function. Emphasis is placed on summarizing the role of mitochondrial metabolic abnormalities in tumor invasion and metastasis, including alterations in the mitochondrial genome (mutations), activation of mitochondrial-to-nuclear signaling, and dynamics within the mitochondria, all intricately linked to the processes of tumor invasion and metastasis. In conclusion, the paper discusses unresolved scientific questions in this field, aiming to provide a theoretical foundation and novel perspectives for developing innovative strategies targeting tumor invasion and metastasis based on mitochondrial biology.

**Graphical Abstract:**

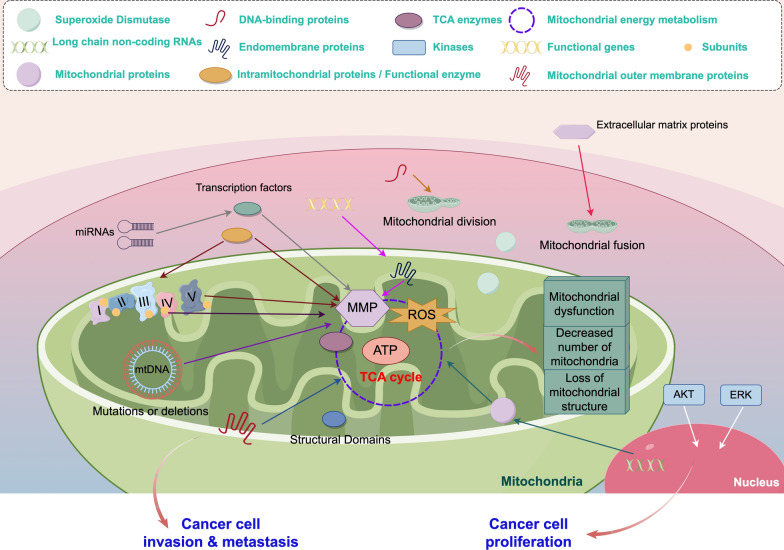

## Mitochondrial structure and function

The mitochondrion is a cellular organelle characterized with a structure comprising two membranes and two aqueous compartments: the outer membrane (OM), intermembrane space (IMS), inner membrane (IM), and matrix. The inner membrane contains the oxidative phosphorylation system, including respiratory complexes I to IV and the F1F0-ATP synthase responsible for ATP production. These hydrophobic membrane proteins form the core components of the mitochondrial inner membrane oxidative phosphorylation complexes,often assembling into complexes and supercomplexes, such as respiratory complexes and protein translocases (Pfanner et al. 2019; Morgenstern et al. 2021; Sung et al. 2020; Rath et al. 2021; Morgenstern et al. 2017; Wittig and Malacarne 2021). Mitochondria serve as the cellular powerhouse, performing various fundamental metabolic processes and regulating cell apoptosis. However, our understanding of the major components of mitochondrial genes and proteins, as well as the biological processes governing the stability and dynamic assembly of mitochondrial proteins, remains limited (Pfanner et al. 2019; Sung et al. 2020; Wittig and Malacarne 2021).

**Fig. 1:**
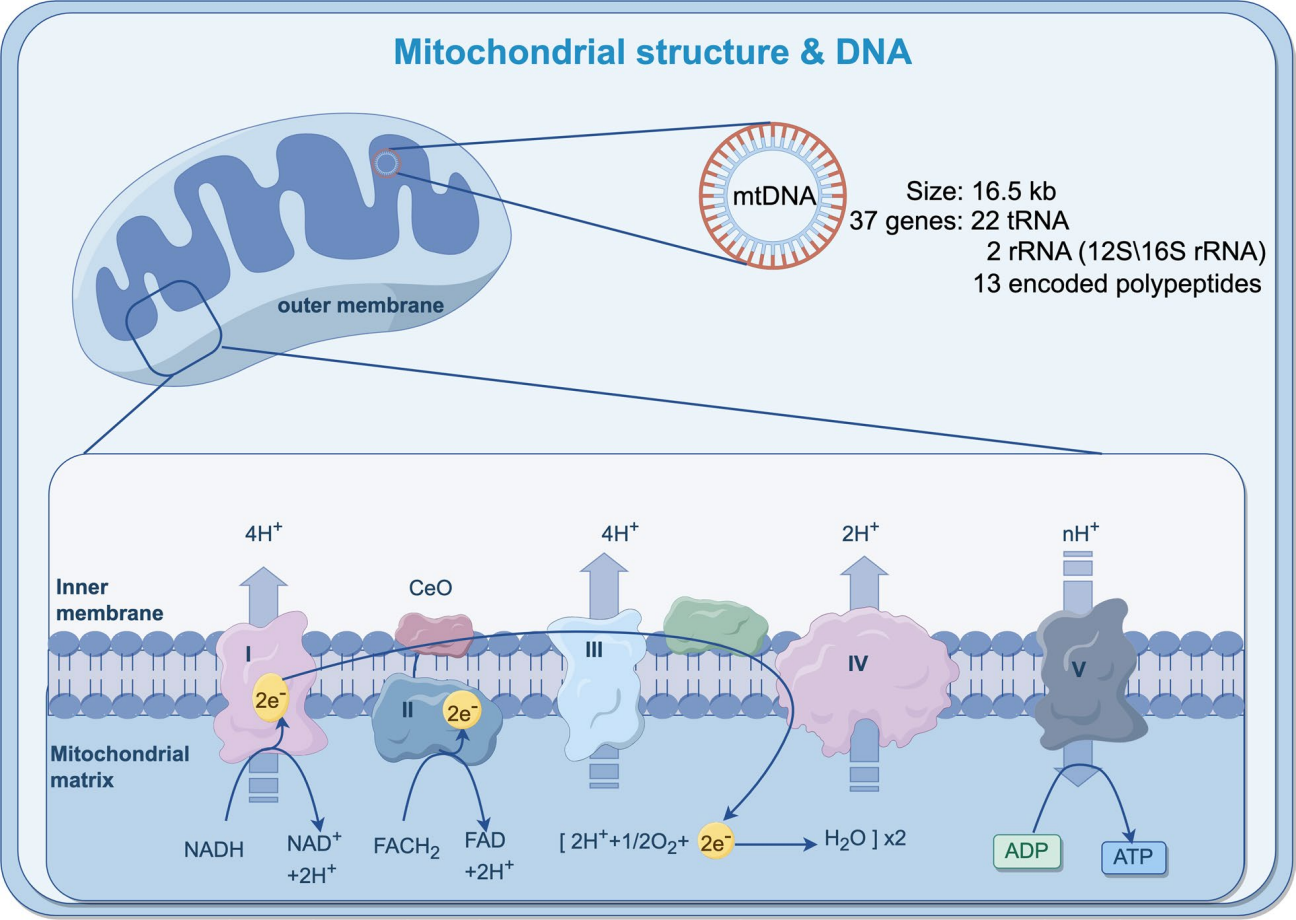
Mitochondrial structure and DNA. Size and content of mitochondrial DNA; ATP production by the mitochondrial respiratory chain. (By Figdraw.)

Human mitochondrial DNA (mtDNA) contains 37 genes, which encode 13 proteins for respiratory complexes I, III, IV, and V (Table 1). Additionally, mtDNA encodes 2 rRNAs and 22 tRNAs necessary for mitochondrial protein synthesis. MtDNA is a circular double-stranded DNA molecule approximately 16.5 kb in length in humans, which consists of a heavy chain (H-chain) rich in G and a light chain (L-chain) rich in C. Notably, the H-chain encodes the majority of mtDNA (Anderson et al. 1981). Furthermore, mtDNA possesses a unique regulatory region known as the displacement loop (D-loop), which includes a short single-stranded DNA molecule, the triple-stranded region 7S DNA (Nicholls and Minczuk 2014). Studies indicate that mutations or deletions in mtDNA can compromise energy metabolism, leading to mitochondrial dysfunction, alterations in intracellular signal transduction, and impact on cellular biological functions. In extreme cases, such alterations can result in "mitochondrial diseases" (Guaragnella et al. 2014; Guerra et al. 2017; Alexeyev et al. 2004; Zeviani and Antozzi 1992). (Fig. 1.)

**Table 1:** The major functions of mitochondrial proteins and their documented associations with specific cancer types

Respiratory complexes	Proteins	Function	Associated illnesses	References
NADH dehydrogenase (complex I)	MT-ND1	Contributes to NADH dehydrogenase activity. Enables protein binding, enables NADH dehydrogenase (ubiquinone) activity	Leber hereditary optic neuropathy (LHON), CRC	Lim et al. 2016; Majander et al. 1991; Xu et al. 2021)
	MT-ND2	Enables protein binding, enables NADH dehydrogenase (ubiquinone) activity, oxidoreductase activity, protein kinase binding, and ionotropic glutamate receptor binding	Leigh syndrome (LS), CRC, GC, BC	Ugalde et al. 2007; Cavalcante et al. 2019; Jayasekera et al. 2023; Li et al. 2015)
	MT-ND3	Enables protein binding and NADH dehydrogenase (ubiquinone) activity	LS, BC	Miller et al. 2014; Martínez-Ramírez et al. 2018)
	MT-ND4	Essential for the catalytic activity and assembly of complex I	LHON, BC,GC, Melanoma, LC	Cavalcante et al. 2019; Vries et al. 1996; Hofhaus and Attardi 1993; Bourges et al. 2004; Bushel et al. 2022; Mahmood et al. 2024; Dasgupta et al. 2012)
	MT-ND4l	Enables NADH dehydrogenase (ubiquinone) activity and oxidoreductase activity, acting on NAD(P)H	LHON	Guo et al. 2017; Brown et al. 1995)
	MT-ND5	Essential for the catalytic activity and assembly of complex I	LHON, LS, Mitochondrial encephalomyopathy with lactic acidosis and stroke-like episodes (MELAS)	Bourges et al. 2004; Liolitsa et al. 2003)
	MT-ND6	Enables NADH dehydrogenase (ubiquinone) activity	LHON, CRC, LC	Vries et al. 1996; Ugalde et al. 2003; Wallace et al. 2016; Yuan et al. 2015)
Coenzyme Q-cytochrome c reductase/cytochrome b (complex III)	MT-CYB	Enables ubiquinol-cytochrome-c reductase activity. Electron transfer activity. Oxidoreductase activity. Enables protein-containing complex binding and metal ion binding	LHON, mitochondrial myopathies,CRC, Prostate cancer,Liver cancer	Wallace et al. 2016; Andreu et al. 1999; Abril et al. 2008; Zhuang et al. 2020)
Cytochrome c oxidase (complex IV)	COX1	Contributes to cytochrome-c oxidase activity. Enables protein binding, heme binding and metal ion binding	LHON, Mitochondrial complex IV deficiency,	Varlamov et al. 2002; Lucioli et al. 2006)
	COX2	Enables cytochrome-c oxidase activity, copper ion binding, protein binding and ion binding	Mitochondrial complex IV deficiency	Power et al. 1989; Rahman et al. 1999)
	COX3	Contributes_to cytochrome-c oxidase activity. Enables protein binding and electron transfer activity	LHON, Mitochondrial complex IV deficiency, Pediatric Malignancies	Johns and Neufeld 1993; Keightley et al. 1996)
ATP synthase	MT-ATP6	Enables protein binding and transmembrane transporter activity. Contributes_to proton-transporting ATP synthase activity,	LHON, LS, Neuropathy, ataxia, and retinitis pigmentosa (NARP), Mitochondrial infantile bilateral striatal necrosis (MIBSN), Mitochondrial complex V deficiency, mitochondrial 1 (MC5DM1), Myopathy, lactic acidosis, and sideroblastic anemia 3 (MLASA3), Ataxia and polyneuropathy, adult-onset (APAO), Cardiomyopathy, infantile hypertrophic (CMHI), CRC, BC, Prostate cancer	Martínez-Ramírez et al. 2018; Wallace et al. 2016; Abril et al. 2008; Aggeler et al. 2002; Tebbenkamp et al. 2018; Holt et al. 1990; Castagna et al. 2007; Vries et al. 1993; Thyagarajan et al. 1995; Rantamäki et al. 2005; Burrage et al. 2014; Craig et al. 2007; Ware et al. 2009; Triska et al. 2019)
	ATP8	Enables protein binding and transmembrane transporter activity. Contributes_to proton-transporting ATP synthase activity	Mitochondrial complex V deficiency, mitochondrial 2 (MC5DM2), Cardiomyopathy, infantile hypertrophic (CMHI), GC, BC,	Aggeler et al. 2002; Ware et al. 2009; Mottaghi-Dastjerdi et al. 2023; Grzybowska-Szatkowska et al. 2014; Thapa et al. 2016)

Mitochondria play a pivotal role in cellular energetics, metabolism, and signal transduction primarily through the generation of adenosine triphosphate (ATP) and intermediate metabolites (Pfanner et al. 2019; Morgenstern et al. 2021; Sung et al. 2020; Nunnari and Suomalainen 2012).Within mitochondria, metabolites produced by the tricarboxylic acid cycle (TCA cycle) and reducing equivalents enter the electron transport chain (ETC). The ETC utilizes oxygen as the terminal electron acceptor to catalyze the oxidation of reducing equivalents. Electron transfer couples with proton translocation across the inner mitochondrial membrane, generating an electrochemical gradient (mitochondrial membrane potential). This gradient, in turn, results in the production of ATP to supply energy to the cellular biological processes. Furthermore, mitochondria serve as a major source of reactive oxygen species (ROS) production within the cell. Consequently, mtDNA is more susceptible to fluctuations in ROS levels, leading to mitochondrial dysfunction and involvement in the processes of diseases such as cancer.

Of particular interest, recent studies demonstrate that mitochondria can undergo intra-cellular migration, maintaining their reticular structure stability through rapid fusion and fission processes. This phenomenon is referred to as mitochondrial dynamics. Mechanistic investigations reveal that a large family of GTPases primarily regulates mitochondrial dynamics. This regulation enables mitochondria to recruit to subcellular compartments requiring additional energy, thereby playing a crucial role in mitochondrial quality control and communication with the cytoplasm and the cell nucleus (Chen and Chan 2009).

Over the past decade, the understanding of the relationship between mitochondria and tumor invasion and metastasis has deepened (Guaragnella et al. 2014; Guerra et al. 2017; Guantes et al. 2015; DeBalsi et al. 2017). Notably, changes in mitochondrial dynamics, such as increased fission and reduced fusion, are commonly observed in clinical tumors and are closely associated with tumor metastasis (Trotta and Chipuk 2017). Furthermore, the instability of mtDNA is closely linked to the occurrence and metastasis of various cancers (Wallace 2012; Choudhury and Singh 2017; Bussard and Siracusa 2017). Recent large-scale analyses of various types of cancer within The Cancer Genome Atlas (TCGA) dataset reveal significant depletion of mtDNA content in tissues of several tumors, including breast cancer (BRCA) and esophageal cancer (ESCA). This depletion leads to reduced expression of mitochondrial respiratory chain genes, showing a negative correlation with the expression of immune response and cell cycle genes (Reznik et al. 2016). These findings indicate that mitochondrial gene expression, protein structures, and their interactions, as well as mitochondrial metabolic abnormalities and dynamic changes, play crucial roles in tumor development, particularly in the invasion and metastasis of tumor cells. This suggests that mitochondria might represent potential novel targets for clinical intervention in cancer. (Table 2).

**Table 2:** Mitochondria-related alterations and related cancers

Mitochondria-related alterations	Tumors	References
Signal transduction	Gastric cancer, liver cancer, lung cancer, cervical cancer, breast cancer, thyroid cancer, colorectal cancer, pancreatic cancer	Frezza and Gottlieb 2009; Liu 2020; Zhao et al. 2013; Chen et al. 2022; Yu et al. 2019; Wan et al. 2021; Lei et al. 2017; Zou et al. 2019)
Gene mutation	Prostate cancer, lung cancer, liver cancer, gastric cancer, colorectal cancer, breast cancer	Ippolito et al. 2019; Grigorian et al. 1996; Chen et al. 2022; Papadaki et al. 2023; Gibellini et al. 2018; Li et al. 2021)
Mitochondrial dynamics	Liver cancer, gastric cancer, breast cancer, lung cancer, colorectal cancer	Lei et al. 2017; Gibellini et al. 2018; Li et al. 2021; Ga et al. 2023; Wu et al. 2023)
Protein & nucleotide expression	Lung cancer, liver cancer, breast cancer, gastric cancer, colorectal cancer, esophagus cancer	Li et al. 2021; Bai and Jiao 2020; Sasaki et al. 2021; Wang et al. 2021; Tang et al. 2018; Lai et al. 2016)

## Mitochondria and cancer invasion and metastasis

### Mitochondria and lung cancer

Lung cancer (LC), one of the most common malignancies, continues to exhibit a rising global incidence and mortality (Sung et al. 2021). Studies reveal that mitochondria play a crucial in the onset and progression of lung cancer. Specifically, abnormalities in the expression of mitochondrial functional genes can trigger a series of complex effects, actively participating in the invasion and metastasis of lung cancer.

Abnormal expression of functional genes can impact mitochondrial function through various mechanisms, including alterations in respiratory chain electron transfer, metabolic remodeling, and mitochondrial dynamics. For instance, overexpression of the NDUFA4 subunit of mitochondrial respiratory chain complex IV alters electron transfer in the mitochondrial respiratory chain, and promotes the growth and migration of human lung cancer cells. This effect is closely linked to the abnormal activation of the AKT and ERK pathways, key drivers of cell proliferation and survival. Real-time PCR assays confirm a significant increase in NDUFA4 expression in lung cancer tissues compared to normal controls, along with corresponding increases in the protein levels of NDUFA4, Akt, p-Akt, Erk, and p-Erk (Lei et al. 2017). The S100A4 protein, also known as metastasin-1 or fibroblast-specific protein-1 (FSP1), is another critical player. It is a well-established marker for epithelial-mesenchymal transition (EMT) and is implicated in tumor metastasis (Grigorian et al. 1996). Liu et al. discover that the mitochondrial complex I subunit NADH dehydrogenase (ubiquinone) Fe-S protein 2 (NDUFS2) is regulated in a S100A4-dependent manner. Cells lacking S100A4 or NDUFS2 undergo a metabolic shift toward glycolysis through upregulation of hexokinase. The S100A4/NDUFS2 axis reshapes mitochondrial metabolism and promotes the invasion and metastasis of lung cancer. Quantitative analysis of oxygen consumption rate (OCR) data reveals that S100A4 knockdown significantly decreases basal respiration, maximal respiration, and spare capacity in lung cancer cells, indicating a predominant effect on mitochondrial respiration (Liu et al. 2019). Zinc finger E-box-binding homeobox 1 (ZEB1), a transcription factor, induce EMT and facilitate metastasis. Bai et al. have revealed that the upregulation of miR-199a-3p leads to the downregulation of ZEB1, subsequently causing mitochondrial dysfunction. This dysfunction includes biological changes such as a decrease in mitochondrial membrane potential, reduced SOD activity, and elevated levels of MDA and LDH, all contributing to the growth and migration of tumor cells (Bai and Jiao 2020).

In addition, mitochondrial abnormalities in ketone metabolism are significant features of cancer cells (Corbet et al. 2018; Li et al. 2017; Lu et al. 2018). Mitochondrial pyruvate carrier 1 (MPC1), located on the inner mitochondrial membrane, is a crucial protein responsible for transporting and oxidizing pyruvate (Herzig et al. 2012; Bricker et al. 2012; McCommis et al. 2016). Zou et al. have revealed that low MPC1 expression in lung adenocarcinoma (LAC) is associated with abnormal mitochondrial function and altered signal transduction. MPC1's role in enhancing pyruvate entry into mitochondrial oxidation and reducing lactate production in tumor cells suggests its impact on tumor cell invasion and metastasis. Further studies examining MPC1 expression in LAC tissues compared to versus adjacent non-tumor tissues corroborate these findings. Additionally, the human PINK1 gene (PTEN-induced kinase 1, Park6), a significant gene in Parkinson's disease, accumulates in the outer mitochondrial membrane and serves as a crucial marker of mitochondrial damage. It triggers autophagy to eliminate depolarized mitochondria. Studies shown that PINK1 knockdown leads to mitochondrial dysfunction, increased ROS generation, and decreased mitochondrial membrane potential, which culminates in suppressed autophagy and affects tumor cell proliferation and migration. Immunohistochemical staining reveals higher PINK1 expression in tumor tissues, which is strongly linked to the tumor-node-metastasis classification and is associated with longer overall survival in non-small cell lung cancer (NSCLC) patients (Zou et al. 2019; Poewe et al. 2017; Schulz et al. 2015; Lu et al. 2020).

It is noteworthy that certain functional genes can also interact with mitochondrial transcriptional regulatory factors. Signal transducer and activator of transcription 3 (STAT3), as a transcription factor, regulates a series of genes related to cancer cell survival, proliferation, angiogenesis, invasion, and metastasis (Song et al. 2011). Studies indicate that the interaction between MPC1 and mitochondrial STAT3 (mito-STAT3) disrupts the distribution of STAT3, reduces the activity of cytoplasmic STAT3 (cyto-STAT3), and promotes the malignant progression of lung cancer, including invasion and metastasis. This suggests the importance of the MPC1/STAT3 axis in the progression of lung cancer and provides a new perspective for molecular targeting of the STAT3 pathway (Zou et al. 2019).

Furthermore, abnormalities in the expression of functional genes can also affect mitochondrial dynamics, contributing to the invasion and metastasis of tumor cells. For example, High Mobility Group Box 1 (HMGB1), belonging to the HMGB superfamily, is a DNA-binding protein that regulates various cellular processes such as inflammation, cell differentiation, and tumor cell migration (Tripathi et al. 2019). Recent studies highlight the crucial role of HMGB1 in tumorigenesis, epithelial-mesenchymal transition, and the prognosis of lung cancer (He et al. 2017). Mechanistically, HMGB1 can induce mitochondrial dynamics dependent on highly phosphorylated dynamin-related protein 1 (DRP1) through the RAGE-extracellular signal-regulated kinase (ERK) signaling pathway (Huang et al. 2018). Liu et al. further found that HMGB1 overexpression increases mitochondrial fission, promoting mitochondrial transport to the leading edge of filopodia through actin filaments and microtubules. This, in turn, enhances cell migration and motility, along with increased expression and phosphorylation of DRP1 in the nucleus and cytoplasm, thereby facilitating lung cancer migration (Liu et al. 2021). Additionally, certain critical structural domains of mitochondrial functional proteins also play important roles. For instance, in invasive lung adenocarcinoma, the expression of Ovarian Cancer Immunoreactive Antigen Domain 2 (OCIAD2) is significantly higher than in situ lung adenocarcinoma, and its abnormal expression correlates with poorer patient prognosis. OCIAD2 is mainly located on the mitochondrial membrane of lung adenocarcinoma cells. Inhibition of OCIAD2 induces a decrease in mitochondrial membrane potential, release of cytochrome c, loss of mitochondrial structure, and a reduction in mitochondrial quantity. Moreover, OCIAD2 inhibition leads to downregulation of cell growth, proliferation, migration, and invasion. Therefore, OCIAD2 may be an effective therapeutic target for lung adenocarcinoma (Hong et al. 2021).

In summary, abnormal expression of mitochondrial genes, including both functional and non-functional genes, can lead to alterations in redox reactions, metabolic reprogramming, and dynamics. These changes contribute to the promotion of invasion and metastasis in lung cancer, suggesting that targeting mitochondrial genes may be a crucial direction for developing novel therapeutic approaches in clinical lung cancer treatment.

### Mitochondria and colorectal cancer

Colorectal cancer (CRC) is one of the most common malignant diseases worldwide and has a high potential for metastasis (Siegel et al. 2020). Recent research suggests that abnormalities in mitochondrial gene expression and function, through changes in energy metabolism, regulate the occurrence of colorectal cancer. For instance, NDUFA4, encoded by the *NDUFA4* gene within mitochondrial respiratory chain complex IV, exhibits significant dysregulation in human CRC and can modulate tumor cell growth and migration, indicating its potential as a novel target for CRC intervention. Similarly, we found high expression of NDUFA4 in human CRC tumor tissues. NDUFA4 overexpression promotes the in vitro growth of human CRC tumor cells, accompanied by alterations in mitochondrial energy metabolism, while downregulation of NDUFA4 expression produces the opposite effect (Liu 2020). Further studies by Liu Shiming et al. revealed that NDUFA4 overexpression can promote the occurrence of epithelial-mesenchymal transition (EMT) in human CRC cells (Liu et al. xxxx). Moreover, Jean Bastin et al. discovered that mitochondrial complex I (CI) is downregulated in the stromal subtype of colorectal cancer (CMS4) cells, which is associated with an increase in mitochondrial reactive oxygen species (mtROS). This suggests that modulating mtROS levels can influence the migration of human CRC cells (Bastin et al. 2023).

It is noteworthy that the changes in the expression of certain functional enzymes can also lead to alterations in mitochondrial function, thereby influencing the invasion and metastasis of colorectal cancer (CRC). For instance, as mentioned earlier, the generation of mitochondrial reactive oxygen species (mtROS) not only occurs through changes in complex I (CI) activity but is also maintained at optimal levels through the inactivation/acetylation of Superoxide Dismutase 2 (SOD2), a major mitochondrial antioxidant enzyme, affecting tumor cell migration (Bastin et al. 2023). Additionally, studies suggest that the Translocase of Outer Mitochondrial Membrane 20 (TOMM20) is overexpressed in various cancers. Sang-Hee Park et al. found that TOMM20, as a receptor for targeting mitochondrial proteins, plays a crucial role in the invasion and metastasis of CRC. Overexpression of TOMM20 increases the proliferation, migration, and invasion of colorectal cancer cells. Mechanistically, TOMM20 expression directly impacts mitochondrial function, including ATP production and membrane potential maintenance, contributing to tumor cell activities such as the regulation of the S-phase cell cycle and apoptosis (Park et al. 2019). Furthermore, Mitochondrial Lon protease (LonP1) is a multifunctional enzyme that regulates mitochondrial function, induces a shift towards glycolysis, and promoting the EMT, thereby conferring migratory and invasive capabilities to tumor cells (Gibellini et al. 2018). Gibellini et al. found that silencing LonP1 leads to severe mitochondrial damage and apoptosis in colon cancer cells. Importantly, LonP1 is virtually absent in normal mucosa, gradually increasing from aberrant crypt foci to adenomas, with the highest expression level in colon cancer. These findings suggest LonP1 is a potential new intervention target for human CRC tumors.

Furthermore, research indicates that non-coding RNAs and transcription factors also play a role in regulating mitochondrial control over the invasion and metastasis of colorectal cancer (CRC) tumor cells. For instance, Wang et al. discovered that the long non-coding RNA FEZF1-AS1 (FEZF1-AS1) is upregulated in colorectal cancer. FEZF1-AS1 regulates the expression of mitochondrial protein phosphoenolpyruvate carboxykinase (PCK2), which is crucial in regulating mitochondrial energy metabolism. This suggests that FEZF1-AS1 deficiency may facilitate the binding of ubiquitin enzymes to PCK2 and promote its degradation. FEZF1-AS1 upregulates PCK2 protein level by inhibiting proteasome-dependent degradation. Knocking out FEZF1-AS1 significantly reduces PCK2 protein level, leading to a decrease in mitochondrial energy metabolism and inhibiting the proliferation and migration of colorectal cancer cells. FEZF1-AS1 is found to increase the protein, but not the mRNA level of PCK2, indicating a post-translational regulatory mechanism (Wang et al. 2021). However, the study does not reveal whether FEZF1-AS1 interacts with PCK2 directly or indirectly, nor does it fully elucidate the mechanisms through which FEZF1-AS1 regulates PCK2 protein levels. Further exploration is needed to uncover the underlying interactions and pathways incolved. Moreover, Lin et al. find that metastatic colorectal cancer cells (SW620) express higher levels of mitochondrial transcription factor A (TFAM) and mtDNA compared to primary SW480 cells. Additionally, the oxygen consumption rate (OCR) and respiratory control ratio (RCR) are higher in SW620 cells. Therefore, it can be concluded that a higher mtDNA copy number and enhanced mitochondrial function may provide an invasion advantage for CRC (Lin et al. 2018).

In summary, aberrant expression of mitochondrial genes can regulate the invasion and metastasis of colorectal cancer, suggesting that targeting mitochondrial-related genes may be a crucial direction for future developments in clinical strategies for colorectal cancer treatment.

### Mitochondria and gastric cancer

Recent studies indicate a close association between the occurrence and invasion-metastasis of gastric cancer (GC) and mitochondrial abnormalities. Sustained communication between the mitochondria and the cell nucleus ensures cellular balance and adaptation to mitochondrial stress.

Compared to normal cells, tumor cells preferentially undergo aerobic glycolysis to metabolize glucose, with several genes encoding enzymes involved in this process prone to alternative splicing (AS). For instance, Vasiliki Papadaki and colleagues discovered that the IQ Motif Containing GTPase Activating Protein 1 (IQGAP1), a scaffold protein, significantly influences mitochondrial respiration by regulating AS in various gene subgroups in GC cells. IQGAP1 promotes tumor development by regulating AS of specific pre-mRNAs related to the cell cycle. Their research shows that IQGAP1 affects AS of components in the electron transport chain (ETC), enhancing oxidative metabolism in GC cells, thus facilitating tumor proliferation and invasiveness (Papadaki et al. 2023). To investigate IQGAP1's role in mitochondrial homeostasis, they examined NUGC4 and NUGC4-IQGAP1KO cells for mitochondrial DNA content, mitochondrial mass, and the expression of transcription factors involved in mitochondrial biogenesis (TFAM, PGC1α, and NRF1). They discovered that IQGAP1 depletion results in a 30% increase in cells with fragmented mitochondrial networks, compared to those with tubular or hyperfused networks. This fragmentation is marked by a decrease in the Aspect Ratio (AR) and Form Factor (FF), indicating reduced mitochondrial elongation and network complexity, along with a lower number of identified mitochondria per cell. Notably, reintroducing cMyc-IQGAP1 in IQGAP1KO cells largely reversed this phenotype. Moreover, IQGAP1 depletion led to a decrease in mitochondrial reactive oxygen species (mtROS) production, which was also restored upon cMyc-IQGAP1 expression. These findings indicate a deficiency in mitochondrial function in the absence of IQGAP1. Additionally, cancer cells generally exhibit higher levels of reactive oxygen species (ROS) compared to normal cells, suggesting that cancer cells are more sensitive to oxidative stress, and mitochondrial reactive oxygen species can accelerate the invasion of GC cells (Chen et al. 2016; Tamura et al. 2014).

In GC cells, the expression of certain nucleic acids also plays a regulatory role. For example, Wu and colleagues found that the overexpression of miR-431-5p significantly inhibits cell proliferation and induces apoptosis, leading to impaired mitochondrial function. This impairment is characterized by a reduction in mitochondrial quantity, a decrease in mitochondrial membrane potential, an increase in mitochondrial permeability transition pore (mPTP) opening, elevated ROS production, and decreased ATP level (Wu et al. 2023). Moreover, increasing evidence suggests that long non-coding RNAs (lncRNAs) can participate in tumorigenesis by regulating invasion, proliferation, and migration (Fonseca Cabral et al. 2020; Lu et al. 2021; Tan et al. 2020; Yang et al. 2022).

Furthermore, recent studies suggest that changes in the expression of certain mitochondrial proteins may impact the development of GC. For instance, NDUFA4 exhibits significantly increased expression in human GC and regulates tumor cell growth and metastasis. NDUFA4 plays a crucial role in GC development by controlling pathways such as mitochondrial oxidative phosphorylation and glycolysis, and regulating other molecules. This highlights its potential value in the prognosis and treatment of GC. To investigate NDUFA4's functions in GC cells, researchers observe its high expression in AGS and HGC27 cells and low expression in MKN45 cells. They found that.the knockdown of NDUFA4 significantly reduced the viability of AGS and HGC27 cells, whereas its overexpression enhanced cell viability in MKN45 cells. Similarly, colony formation was significantly inhibited by shNDUFA4 but promoted by overexpressed NDUFA4 (Frezza and Gottlieb 2009; Gogvadze et al. 2008; Li et al. Dec 2018; Zhang et al. 2019; Xu et al. 2022; Cheng et al. 2012). Additionally, small molecules can influence mitochondrial metabolism to regulate tumor invasion and metastasis. For example, Yuto Sasaki and colleagues discovered that asporin (ASPN) is a leucine-rich small proteoglycan mainly expressed by cancer-associated fibroblasts (CAFs), playing a crucial role in tumor progression. ASPN expression enhances GC cell resistance to oxidative stress by reducing mitochondrial ROS. ASPN induces the expression of the transcription factor HIF1α and upregulates lactate dehydrogenase A (LDHA), indicating that ASPN reprograms GC cells to undergo anaerobic glycolysis, suppressing ROS generation in the mitochondria. Overexpression of ASPN in two GC cell lines leads to increased migration and invasion capabilities (Sasaki et al. 2021).

Similarly, the changes in the expression of certain functional enzymes can regulate the invasion and metastasis of tumor cells. For example, Mi Y and colleagues confirmed a significant increase in the expression of mitochondrial creatine kinase (uMtCK) in GC tissues, which is significantly associated with poorer prognosis, especially in advanced-stage patients. Functionally, uMtCK promotes glycolysis in GC cells and enhances their migration, invasion, and liver metastasis in both in vitro and in vivo settings. Mechanistically, uMtCK enhances the occurrence and invasion-metastasis of GC through the JNK-MAPK/JUN signaling pathway, thereby reinforcing HK2-dependent glycolysis Mi et al. 2023.

In summary, the expression levels of mitochondrial functional genes are closely associated with the metastasis of GC, making them potential targets for intervention. A comprehensive understanding of these molecular mechanisms could contribute to the development of novel anti-cancer strategies targeting mitochondria.

### Mitochondria and breast cancer

Breast cancer (BC) is the most common cancer among women globally, posing a significant threat to women's health. Investigating its biological mechanisms is crucial for improving patient prognosis.

The metabolic imbalance of mitochondria is closely associated with the growth and metastasis of breast cancer. In epithelial breast cancer cells, the oxidative phosphorylation (OxPhos) level is upregulated, enabling these cells to generate high levels of ATP, thereby promoting proliferation and invasive metastasis (Nayak et al. 2018). This metabolic imbalance is often correlated with changes in the expression of related genes. For example, Wang et al. identified an upregulation of citrate synthase (ACLY) in breast cancer. ACLY is the first crucial enzyme in the production of acetyl-CoA, and its overexpression is associated with pathological grading, tumor size, and lymph node metastasis. Inhibiting ACLY can effectively suppress tumor growth (Wang et al. 2017). The expression of mitochondrial ribosomal protein L52 (MRPL52) is elevated in human breast cancer and significantly correlated with invasive clinical pathological features and higher metastatic risk. Li et al. found that MRPL52 overexpression in breast cancer is induced by hypoxia-inducible factor 1 in response to hypoxic exposure, demonstrating its role in inhibiting apoptosis and promoting migration and invasion of hypoxic breast cancer cells (Li et al. 2021).

Triple-negative breast cancer (TNBC) is an aggressive form of breast cancer. The tumor microenvironment, rich in pro-inflammatory cytokines like TNF-α, regulates cancer cells' bioenergetic capacity, immune evasion, and survival. NLRX1, a mitochondrial NOD-like receptor protein, regulates mitochondrial function during apoptosis and tissue damage. Depletion of NLRX1 impairs lysosomal function, leading to altered turnover of damaged mitochondria through mitophagy in the presence of TNF-α. The loss of NLRX1 reduces OxPhos-dependent cell proliferation and migration under TNF-α conditions, supporting the tumorigenic potential of invasive breast cancer cells by maintaining energy homeostasis and preserving organelle functio (Singh et al. 2015; Singh et al. 2019).

Dynein Light Chain Tctex-Type 1 (DYNLT1) is upregulated in breast tumors and is upregulated in breast tumors and is a crucial component of the motor complex that transports cellular cargo along microtubules. DYNLT1 co-localizes with Voltage-Dependent Anion Channel 1 (VDAC1) on the mitochondria, regulating essential metabolic and energy functions, thereby promoting proliferation, migration, invasion, and mitochondrial metabolism of breast cancer cells in vitro, and facilitating the development of breast tumors *in vivo* (Huang et al. 2023). Epidermal Growth Factor-Like 9 (EGFL9) is significantly upregulated in basal-like breast cancer cells and is associated with the metastatic progression of breast tumor samples. EGFL9 is both necessary and sufficient to enhance cancer cell migration, invasion, and distant metastasis (Meng et al. 2019). NDUFA4 is also overexpressed in breast cancer, leading to enhanced oxidative phosphorylation and increased ATP consumption (Li et al. 2020).

Changes in gene expression can lead to alterations in signaling pathways and mitochondrial dynamics. For example, Li et al. found that mitochondrial ribosomal protein L52 (MRPL52) enhances epithelial-mesenchymal transition, migration, and invasion of hypoxic breast cancer cells by activating the ROS-Notch1-Snail signaling pathway (Li et al. 2021). Another study revealed a significant upregulation of mitochondrial fission protein Dynamin-related protein 1 (DRP1) in human invasive breast cancer and lymph node metastasis. Compared to non-metastatic breast cancer cells, metastatic cells exhibited more fragmented mitochondria, higher levels of total and active DRP1, and lower expression of mitochondrial fusion protein 1 (Mfn1). Silencing DRP1 or overexpressing Mfn1 led to mitochondrial elongation or clustering, respectively, significantly inhibiting the metastatic ability of breast cancer cells (Zhao et al. 2013). Human MARCH 5, a mitochondria-localized E3 ubiquitin ligase, promotes the growth and metastasis of breast cancer cells in vitro and in vivo.,mainly mediated through increased mitochondrial fission and subsequent ROS production (Tang et al. 2019).

In summary, the studies mentioned above have revealed the crucial role of mitochondria in the growth and metastasis of breast cancer cells. This provides a significant foundation for further developing clinical treatment strategies targeting specific genes within the mitochondria for breast cancer.

### Mitochondria and liver cancer

Recent studies have found that many mitochondria-related proteins can influence the growth and metastasis of liver cancer cells, participating in the development of hepatocellular carcinoma (HCC). For instance, mitochondrial transcription elongation factor (TEFM) is a novel nuclear-encoded factor involved in mitochondrial genome transcription. Fei et al. found that both protein and mRNA expression levels of TEFM were significantly upregulated in HCC tissues compared to non-cancerous liver tissues (Zy et al. 2020). Additionally, TEFM mRNA expression levels were significantly associated with vascular invasion. Further analysis revealed that high expression levels of TEFM were associated with poor prognosis in HCC patients. Wan et al. also found that TEFM partially exerted its tumor-promoting effects by increasing ROS production and subsequently activating the ERK signaling pathway. Increased TEFM expression in HCC tissues was mainly attributed to the downregulation of miR-194-5p. TEFM upregulation was associated with poor prognosis in HCC patients (Wan et al. 2021). Recently, phosphodiesterase 2A (PDE2A) has been found to be involved in the regulation of mitochondrial function andclosely associated with the progression of various types of tumors. For example, Chen et al. found that overexpression of PDE2A could inhibit proliferation, colony formation, migration, and invasion of two HCC cell lines, while inhibition of PDE2A had the opposite effect. The mechanism of action of PDE2A on HCC cells is attributed to changes in mitochondrial morphology and ATP levels (Chen et al. 2022).

Similarly, changes in the expression of some mitochondrial functional proteins can also affect tumor development. For instance, Zhang et al. found high expression of the mitochondrial translocator protein (TSPO) in HCC, which is associated with poor prognosis. Mitochondrial TSPO promotes the growth, migration, and invasion of HCC cells by inhibiting ferroptosis ( A type of regulated cell death characterized by excessive ROS‐mediated lipid peroxidation, which eventually leads to plasma membrane damage and cell death (Stockwell 2022).)and anti-tumor immune responses (Zhang et al. 2023). Notably, the expression of NDUFA4 complex molecule NDUFA4L2 is abnormal in HCC and plays a role in regulating tumor cell growth and metastasis (Lai et al. 2016; Tello et al. 2011).

Certainly, dysregulation of mitochondrial dynamics is closely associated with tumorigenesis. For example, Tian et al. found that the extracellular matrix-related protein CCBE1 promotes mitochondrial fusion in HCC. They observed a significant downregulation of CCBE1 expression in tumors compared to non-tumor tissues, which was attributed to the high methylation of the CCBE1 promoter in HCC (Ga et al. 2023). Furthermore, overexpression of CCBE1 or treatment with recombinant CCBE1 protein significantly inhibited the proliferation, migration, and invasion of HCC cells both in vitro and in vivo. Huang et al. discovered that increasing mitochondrial division by forced expression of DRP1 or knockdown of MFN1 promotes the survival and invasion of HCC cells in vitro and *in vivo* (Huang et al. 2016). Moreover, Glia Maturation Factor-β (GMFB) is known as a growth and differentiation factor for glial cells and neurons. In the study by Wan Sun et al., GMFB expression was significantly upregulated in HCC patients and positively correlated with tumor node metastasis (TNM) stage and histological grade of HCC. Deletion of GMFB in cancer cells significantly inhibited proliferation, migration, and invasion of cancer cells, downregulated the expression levels of some matrix metalloproteinases (MMPs), and increased mtDNA copy number and loss of mitochondrial transmembrane potential (Sun et al. 2021).

In general, abnormal expression of mitochondrial-related genes may lead to changes in oxidative-reduction reactions, metabolic reprogramming, and mitochondrial dynamics, thereby promoting the invasion and metastasis of liver cancer. This suggests that targeting mitochondrial genes or proteins, and further elucidating their mechanisms, could provide a new perspective for mitochondria-targeted therapy in liver cancer.

### Mitochondria and other cancers (esophageal, thyroid, pancreatic, prostate, cervical)

Prostate Cancer (PCa) is the most common malignant tumor in the male reproductive system. In addition to mtDNA mutations/deletions found in cancer cells, mutations in nuclear-encoded mitochondrial enzymes are also associated with tumorigenesis (Thompson 2009). These findings suggest that tumorigenesis occurring in mitochondria involves not only defects in mitochondrial energy production but also changes in mitochondrial biogenesis and metabolism. Regarding PCa, the invasive phenotype of cancer cells is related to metabolic shifts toward aerobic glycolysis, citrate oxidation, and loss of zinc accumulation (Shiraishi et al. 2015). CAFs are a major cellular stromal component in many solid tumors. CAFs establish a metabolic symbiosis with PCa cells, enhancing cancer invasiveness through lactate shuttle mechanisms (Ippolito et al. 2019). Importantly, metabolites are not only exchanged from CAFs to cancer cells in soluble form but also via exosome shuttle. In this scenario, amino acids and TCA cycle intermediates can upregulate glycolysis while downregulating OxPhos, contributing to tumorigenesis and invasion (Zhao et al. 2016). Furthermore, mutations in mitochondrial-encoded respiratory complex I are significantly associated with PCa. Mutations in respiratory complex I can lead to decreased expression levels of the corresponding complex in prostate cancer, increased expression of MFN1, MFN2, and PINK1, and decreased expression of MT-TFA, thereby promoting PCa cell cycle progression and invasiveness. Philley et al. suggest that mutations in complex I are associated with increased mitochondrial fusion (Philley et al. 2016).

Pancreatic cancer is the third leading cause of cancer-related deaths. Increasing evidence suggests that mitochondrial metabolites may act as second messengers, inducing epigenetic changes in the nucleus (Frezza 2017; Shaughnessy et al. 2014). Acetyl-CoA is one of the most extensively studied signaling molecules. Recent work has reported that overexpression of ACLY leads to elevated levels of acetyl-CoA in the cytoplasmic/nuclear, which promotes the invasiveness of pancreatic cancer (Carrer et al. 2019). Depletion of DRP1 in pancreatic cancer cells has been shown to reduce oxygen consumption rates and ATP production levels, leading to decreased tumor growth and metastasis *in vivo* (Yu et al. 2019). Studies have shown that pancreatic tumors exhibit metabolic heterogeneity and contain cancer stem cell subpopulations with high metastatic and tumorigenic potential, which rely on OxPhos (Viale et al. 2015). Recently, studies have found that NDUFA4 is upregulated in pancreatic cancer tissues, and its high expression levels are negatively correlated with patient survival rates. Zhang Y further discovered that downregulation of NDUFA4 induces G1 phase arrest, reducing proliferation in human pancreatic cancer cells. This downregulation of NDUFA4 significantly inhibits tumor growth and invasion *in vivo* (Zhang et al. 2022).

Cervical cancer is the fourth most common cancer in women. When diagnosed early and treated effectively, it is one of the most treatable cancers. Xin et al. found that ACLY is upregulated in cervical cancer, which can enhance the proliferation and invasion of cervical cancer cells (Xin et al. 2016). Recent research has identified Double C-2 like domain beta (DOC2B) as a tumor suppressor, exhibiting anti-proliferative, anti-migratory, anti-invasive, and anti-metastatic functions in cervical cancer. Divya Adiga et al. confirmed the tumor growth-regulating function of the DOC2B-mitochondrial axis in cervical cancer. They found that DOC2B expression induces changes in mitochondrial morphology, accompanied by a decrease in mitochondrial DNA copy number, mitochondrial mass, and mitochondrial membrane potential. In the presence of DOC2B, intracellular and mitochondrial levels of Ca_2_^+^, intracellular O.-_2_, and ATP are significantly increased, while glucose uptake, lactate production, and mitochondrial complex IV activity are reduced. AMPK signaling is also activated. These changes ultimately promote the invasion and metastasis of cervical cancer (Adiga et al. 2023).

Thyroid cancer is a malignant tumor originating from the follicular epithelial cells of the thyroid gland or parafollicular cells and is the most common malignant tumor in the head and neck region. Although evidence suggests a correlation between mutations in mtDNA and nDNA, Notably, approximately 20% of patients exhibit mitochondrial gene mutations without concurrent nuclear gene mutations, indicating that some mtDNA mutations may be involved in carcinogenesis (Yuan et al. 2020). Correlations between mitochondrial and nuclear mutation burdens have been observed in various types of cancers, including an increase in thyroid cancer. Furthermore, deleterious mitochondrial mutations are associated with the overexpression of genes involved in cancer signaling pathways, such as the TNFα pathway, OxPhos, and protein secretion pathways (Yuan et al. 2020). Papillary thyroid carcinoma (PTC) is the most common malignant tumor of the thyroid gland. Bojie Chen et al. detected cyclin D1 and mitochondrial complex IV in PTC patients with tumor lymph node metastasis (LNM) samples. They found that downregulation of mitochondrial function negatively affects tumor progression and LNM through the PI3K/Akt/FoxO1/Cyclin D1 pathway (Chen et al. 2022).

Esophageal cancer originates from the squamous epithelium and columnar epithelium of the esophagus and is a common malignant tumor of the digestive tract. In China, esophageal squamous cell carcinoma (ESCC) is the most common type. Recent research has found that the expression level of NDUFA4 in ESCC tissues is significantly lower than that in adjacent non-cancerous tissues. The expression of NDUFA4 is closely associated with the clinical stage, depth of infiltration, histological grade, and lymph node metastasis of ESCC patients. Studies have shown that NDUFA4 regulates tumor cell growth and metastasis in ESCC through the interactions with other molecules. For example, Tang et al. found that NDUFA4 is a direct target of miR-147b. The expression of NDUFA4 in ESCC tissues is negatively correlated with the expression of miR-147b. Inhibitors of miR-147b significantly increase the expression of NDUFA4 in ESCC EC1 and EC9706 cells, inhibiting the proliferation and invasion ability of ESCC, and altering the distribution of the cell cycle (Tang et al. 2018).

**Fig. 2:**
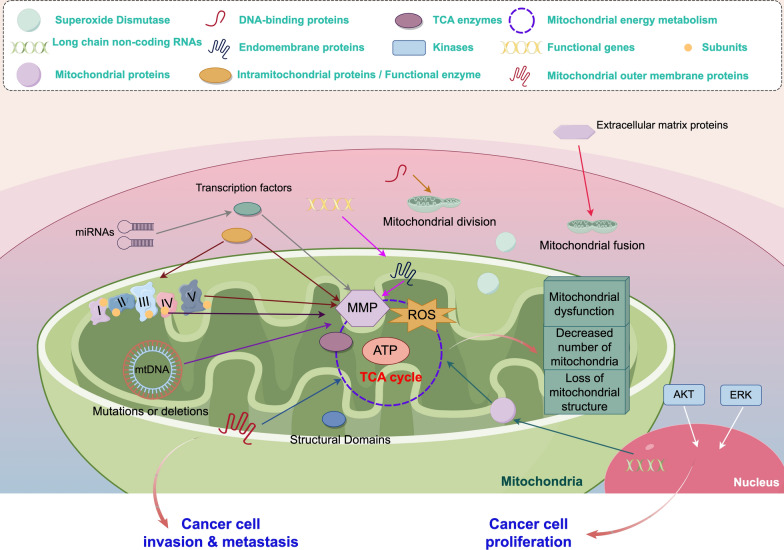
Mitochondrial cancer-related mechanisms. Mitochondrial energy metabolism and mitochondrial dynamics and alterations in mitochondrial structure, number, and function ultimately lead to tumor invasion and metastasis. (By Figdraw.)

In conclusion, the regulation of mitochondrial gene expression plays a crucial role in the development and progression of cancer, highlighting its potential value in prognosis assessment and treatment of cancer. (Fig. 2.)

### Advances in research and clinical practice of mitochondrial drug therapy

Over the past decade, significant progress has been made in the study of mitochondrial-targeted drugs. In cancer cells, alterations in mitochondrial signaling and metabolic pathways are frequently observed, contributing to cancer development and progression. These changes render cancer cells more susceptible to mitochondrial-targeted therapies. Loss of functional mitochondria can lead to stagnation of cancer progression or cell death.

Currently, research on drugs targeting mitochondria mainly focuses on several key aspects. Among them, the biguanide class of antidiabetic drugs (such as metformin and phenformin) is one of the most extensively studied. Although their mechanisms are not fully understood, it is confirmed that these drugs inhibit electron transport chain complex I (NADH dehydrogenase) (Ashton et al. 2018; Chae and Kim 2018; García Rubiño et al. 2019; Andrzejewski et al. 2018; Luengo et al. 2014). These drugs may affect the survival capability of cancer cells by inducing mitochondrial respiratory dysfunction, reducing ATP synthesis, and mediating ROS-induced apoptosis. A recent study on pancreatic cancer reported that metformin is toxic to undifferentiated tumor cells with high mitochondrial content. This toxicity is attributed to the bioenergetic stress caused by the inability of cells to activate glycolysis after OxPhos inhibition (Deschênes-Simard et al. 2019). Additionally, metformin can inhibit the self-renewal ability and tumorigenicity of osteosarcoma stem cells by directly targeting mitochondria, resulting in reduced ATP synthesis and ROS-mediated apoptosis (Zhao et al. 2019). In other types of cancer, metformin can increase sensitivity to chemotherapy drugs, reduce the migratory ability of drug-resistant cells, decrease the number of cancer stem cells, and inhibit tumor proliferation and angiogenesis (Bishnu et al. 2019; Markowska et al. 2018). However, some types of cancer cells may develop resistance, necessitating combination therapy or the use of more potent inhibitors. Studies have shown that treatment of breast cancer cells with low concentrations of metformin may induce metabolic reprogramming in cancer stem cells by increasing glycolysis to acquire drug resistance. Nevertheless, such metabolic changes also offer opportunities for other therapeutic approaches (Banerjee et al. 2019). Such as conjugating the lipophilic cation triphenylphosphonium (TPP +) with metformin can selectively target mitochondria in animal models of pancreatic cancer, thereby enhancing therapeutic efficacy (Kalyanaraman et al. 2018).

In addition to the biguanide class of antidiabetic drugs, other compounds targeting mitochondrial complexes include menadione and Pirvinium pamoate (Diehn et al. 2009; Lamb et al. 2015). These drugs induce the generation of ROS and help prevent cancer cells from developing resistance. Furthermore, resveratrol, through its actions on complexes I and IV, reduces growth rates and invasion potential in various cancer cell lines (Lopes Costa et al. 2014; Wang and Moraes 2011). Additionally, this molecule induce mitochondrial dysfunction, cytochrome c release, and caspase activation in pancreatic cancer (Xu et al. 2015).

Furthermore, drugs like oligomycin and resveratrol have demonstrated inhibitory effects on cancer cell growth and invasion. Oligomycin is a well-known inhibitor of ATP synthase. Although its toxicity precludes its use as a single therapy for cancer treatment, it shows efficacy when used in combination with drugs like 2-DG or nicotinamide against cancer stem cells in glioblastoma, ovarian cancer, and breast cancer cells (Yo et al. 2012; Wang et al. 2013). Salinomycin, on the other hand, is a K^+^ ionophore and uncoupling agent that inhibits OxPhos. It reduces the percentage of tumor-initiating cells (TICs) both in vitro and in vivo across various types of cancer (Naujokat and Steinhart 2012; Jangamreddy et al. 2015; Jiang et al. 2018). Moreover, salinomycin overcome drug resistance and sensitizes tumor cells to radiotherapy (Hermawan et al. 2016; Ko et al. 2016; Qi et al. 2022; Dewangan et al. 2017; Magrath et al. 2020).

In clinical trials, mitochondrial drugs are typically used in combination with traditional cancer treatment such as chemotherapy and radiotherapy. Additionally, the combined use of uncoupling agents and other mitochondrial-targeted drugs is being evaluated. These studies aim to enhance tumor cell sensitivity to treatment and reduce treatment therpy (Zhang et al. 2014; Jara et al. 2014; Zhang et al. 2015). Lapatinib, a dual inhibitor of epidermal growth factor receptor (EGFR) and ErbB2, with anti-proliferative effects, has been used to treat metastatic breast cancer patients with ErbB2 overexpression (Burris 2004).

Overall, the advancements in mitochondrial drug therapy research provide new directions and hope for cancer treatment. However, further research is still needed to understand the mechanisms of mitochondria in cancer development to develop more effective treatment drugs and strategies.

## Summary and prospects

Metabolic changes play a crucial role in the growth and metastasis of tumor cells, Meanwhile, mitochondria serve as the primary organelles for energy metabolism. Studies have demonstrated the intimate association between mitochondrial functional gene reprogramming, alterations in respiratory chain transmission, and dynamic anomalies with tumor occurrence. This research underscores the significance of mitochondrial metabolism in tumor biology, highlighting the complexity of tumor metabolic remodeling and emphasizing mitochondria as a critical new target for future clinical intervention in tumors.

However, novel mitochondrial-based strategies for tumor intervention still face several key issues that urgently need clarification:

First, further studies on the precise mechanisms of mitochondrial mtDNA gene expression are urgently needed. The regulation of mitochondrial gene expression differs from that of nuclear genes. Abnormal mitochondrial gene expression can lead to changes in redox reactions, metabolic reprogramming, and dynamics. It remains to be claried which mitochondrial genes directly impact ATP synthesis and potentially regulate processes like cancer invasion and metastasis. Furthermore, the mechanisms underlying changes in mitochondrial gene copy numbers remain to be elucidated. It is essential to investigate whether alterations in copy numbers are associated with cancer invasion and metastasis. Finally, the exact relationship among changes in mitochondrial dynamics, gene expression and cell migration, as well as related signal transduction pathways affect this process, also requires further study.

Second, the relationship between mitochondrial metabolic reprogramming in tumor cells and other metabolic processes, the complex relationship among carbohydrate, lipid, protein, nucleic acid metabolism with mitochondrial metabolism, needs further elucidation. For example, synergistic or competitive relationships between glycolysis and mitochondrial oxidative phosphorylation in the sugar metabolism pathway may affect the cell's energy utilization. Furthermore, abnormalities in lipid metabolism may also affect mitochondrial function and metabolic pathways, thereby affecting cell growth and metastatic potential (Shao et al. 2024). Therefore, research on the interactions and regulatory mechanisms will enhance our understanding of tumor cell metabolism, offering new avenues and insights for developing therapeutic strategies targeting tumor metabolism.

**Fig. 3:**
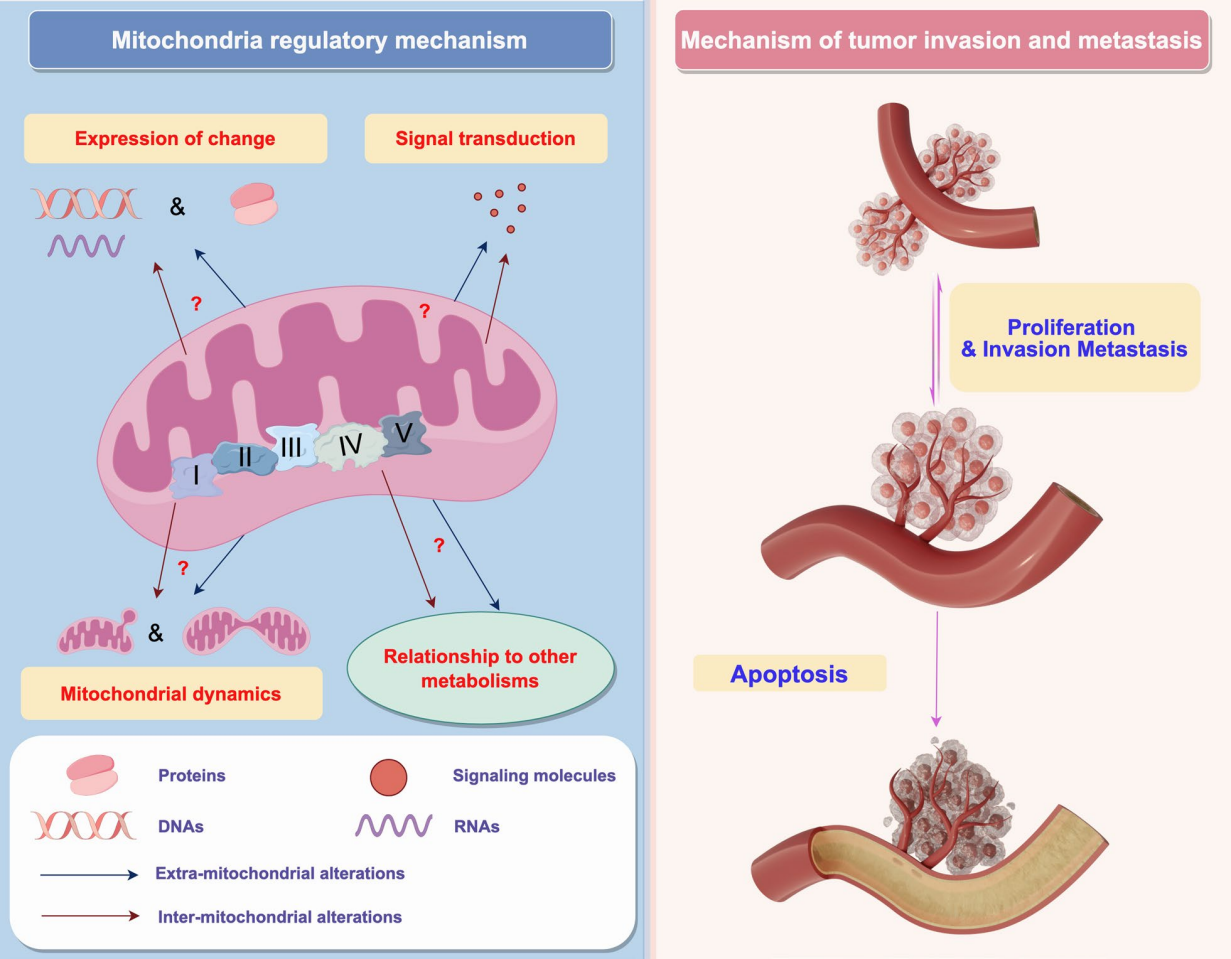
Prospects of mitochondrial explorations. Focusing on 4 mitochondria related to tumor invasion and metastasis for outlook to target mitochondria to control tumor apoptosis. (By Figdraw.)

Finally, the relationship between changes in mitochondrial function and tumor growth and metastasis needs further investigation. For instance, recent discoveries have linked cell ferroptosis to tumor cell growth and metastasis, with changes in mitochondrial iron pools participating in ferroptosis. However, the intrinsic connection between this process and changes in mitochondrial metabolism,as well as the relationship between ferroptosis and tumor growth and metastasis, still requires exploration. Additionally, tumor stem cells play a crucial role in tumor growth and metastasis, and understanding the relationship between mitochondrial metabolic abnormalities and tumor stem cells, along with the underlying mechanisms, is essential for further investigation. (Fig. 3.)

In conclusion, the emerging applications of mitochondrial-targeted drugs in clinical trials, highlight the promising potential of mitochondria-targeted therapy. Recent technological advancement have greatly improved our understanding of mitochondria and their role in tumor biology. High-throughput sequencing has enabled a comprehensive analysis of mtDNA mutations, increasingly recognized as critical factors in cancer development (Hong et al. 2020). Live-cell imaging techniques have provided real-time insights into mitochondrial dynamics, such as fusion and fission, within living cells (Alieva et al. 2023; Dou et al. 2023). Furthermore, the CRISPR/Cas9 system has genetic editing, allowing for targeted manipulation of mitochondrial genes to study their effects on tumor development (Liu et al. 2023; Wang et al. 2022; Dekkers et al. 2020). These technologies have opened new avenues for investigating the intricate relationship between mitochondria and cancer. With the rapid advancement of molecular and cellular biology technologies, including single-cell sequencing, spatial transcriptomics, spatiotemporal transcriptomics, and metabolomics, we can further elucidate the relationship between mitochondria and tumor development as well as the underlying mechanisms. This will provide robust support for the development of novel strategies targeting mitochondria for tumor gene therapy, ultimately improving clinical treatment outcomes for tumors. Through in-depth research into the role of mitochondria in tumor development, we can better understand the biological characteristics of tumors, offering moreopportunities for personalized and precision medicine.

## Data Availability

No datasets were generated or analysed during the current study.

## Abbreviations

AS: Alternative splicing
APAO: Ataxia and polyneuropathy, adult-onset
BC: Breast cancer
CAFs: Cancer-associated fibroblasts
CMHI: Cardiomyopathy, infantile hypertrophic
CRC: Colorectal cancer
DOC2B: Double C-2 like domain beta
DYNLT1: Dynein Light Chain Tctex-Type 1
EGFL9: Epidermal Growth Factor-Like 9
EGFR: Epidermal growth factor receptor
EM: Energy metabolism
EMT: Epithelial-mesenchymal transition
ETC: Electron transport chain
ESCC: Esophageal squamous cell carcinoma
FEZF1-AS1: Long non-coding RNA FEZF1-AS1
FSP1: Fibroblast-specific protein-1
GC: Gastric cancer
GMFB: Glia Maturation Factor-β
HCC: Hepatocellular carcinoma
HMGB1: High Mobility Group Box 1
IMS: Intermembrane space
IQGAP1: IQ Motif Containing GTPase Activating Protein 1
LAC: Lung adenocarcinoma
LC: Liver cancer
LDHA: Lactate dehydrogenase A
Leigh Syndrome (LS): Leigh syndrome
LHON: Leber hereditary optic neuropathy
LonP1: Mitochondrial Lon protease
lncRNAs: Long non-coding RNAs
mPTP: Mitochondrial permeability transition pore
MRPL52: Mitochondrial ribosomal protein L52
MPc1: Mitochondrial pyruvate carrier 1
mtDNA: Mitochondrial DNA
mtROS: Mitochondrial reactive oxygen species
MT-CYB: Mitochondrial complex V deficiency, mitochondrial 1 (MC5DM1)
MLASA3: Myopathy, lactic acidosis, and sideroblastic anemia 3
MELAS: Mitochondrial encephalomyopathy with lacticacidosis and stroke-like episodes
NAP: Neuropathy, ataxia, and retinitis pigmentosa
MIBSN: Mitochondrial infantile bilateral striatal necrosis
NDUFA4: NADH dehydrogenase 1 alpha subcomplex 4
NDUFA4L2: NDUFA4 mitochondrial complex 2
NDUFS2: Mitochondrial complex I subunit NADH dehydrogenase (ubiquinone) Fe-S protein 2
OCR: Oxygen consumption rate
OM: The outer membrane
PAAD: Pancreatic adenocarcinoma
PCK2: Mitochondrial protein phosphoenolpyruvate carboxykinase
PINK1: PTEN-induced kinase 1
PTC: Papillary thyroid carcinoma
RCC: Renal cell carcinoma
SOD2: Superoxide Dismutase 2
STAT3: Signal transducer and activator of transcription 3
TBS: Triple-negative breast cancer
TefM: Mitochondrial transcription elongation factor
TFAM: Mitochondrial transcription factor A
TGCA: The Cancer Genome Atlas
TPP+: Triphenylphosphonium
TICs: Tumor-initiating cells
TOMM20: Translocase of Outer Mitochondrial Membrane 20
TSPO: Mitochondrial translocator protein
uMtCK: Mitochondrial creatine kinase
VDAC1: Voltage-Dependent Anion Channel 1

## References

[CR1] Abril J, de Heredia ML, González L, et al. Altered expression of 12S/MT-RNR1, MT-CO2/COX2, and MT-ATP6 mitochondrial genes in prostate cancer. Prostate. 2008;68(10):1086–96. 10.1002/pros.20771. 18409190 10.1002/pros.20771

[CR2] Adiga D, Bhat S, Shukla V, et al. Double C-2 like domain beta (DOC2B) induces calcium dependent oxidative stress to promote lipotoxicity and mitochondrial dysfunction for its tumor suppressive function. Free Radic Biol Med. 2023;201:1–13. 10.1016/j.freeradbiomed.2023.03.010. 36913987 10.1016/j.freeradbiomed.2023.03.010

[CR3] Aggeler R, Coons J, Taylor SW, et al. A functionally active human F1F0 ATPase can be purified by immunocapture from heart tissue and fibroblast cell lines. Subunit structure and activity studies. J Biol Chem. 2002;277(37):33906–12. 10.1074/jbc.M204538200. 12110673 10.1074/jbc.M204538200

[CR4] Alexeyev MF, Ledoux SP, Wilson GL. Mitochondrial DNA and aging. Clin Sci. 2004;107(4):355–64. 10.1042/cs20040148. 10.1042/cs20040148 15279618

[CR5] Alieva M, Wezenaar AKL, Wehrens EJ, Rios AC. Bridging live-cell imaging and next-generation cancer treatment. Nat Rev Cancer. 2023;23(11):731–45. 10.1038/s41568-023-00610-5. 37704740 10.1038/s41568-023-00610-5

[CR6] Anderson S, Bankier AT, Barrell BG, et al. Sequence and organization of the human mitochondrial genome. Nature. 1981;290(5806):457–65. 10.1038/290457a0. 7219534 10.1038/290457a0

[CR7] Andreu AL, Hanna MG, Reichmann H, et al. Exercise intolerance due to mutations in the cytochrome b gene of mitochondrial DNA. N Engl J Med. 1999;341(14):1037–44. 10.1056/nejm199909303411404. 10502593 10.1056/nejm199909303411404

[CR8] Andrzejewski S, Siegel PM, St-Pierre J. Metabolic profiles associated with metformin efficacy in cancer. Front Endocrinol. 2018;9:372. 10.3389/fendo.2018.00372. 10.3389/fendo.2018.00372 PMC6110930 30186229

[CR9] Ashton TM, McKenna WG, Kunz-Schughart LA, Higgins GS. Oxidative phosphorylation as an emerging target in cancer therapy. Clin Cancer Res. 2018;24(11):2482–90. 10.1158/1078-0432.Ccr-17-3070. 29420223 10.1158/1078-0432.Ccr-17-3070

[CR10] Bai J, Jiao WY. Down-regulation of ZEB1 by miR-199a-3p overexpression restrains tumor stem-like properties and mitochondrial function of non-small cell lung cancer. Onco Target Therapy. 2020;13:4607–16. 10.2147/ott.S244525. 10.2147/ott.S244525 PMC7250308 32547091

[CR11] Banerjee A, Birts CN, Darley M, et al. Stem cell-like breast cancer cells with acquired resistance to metformin are sensitive to inhibitors of NADH-dependent CtBP dimerization. Carcinogenesis. 2019;40(7):871–82. 10.1093/carcin/bgy174. 30668646 10.1093/carcin/bgy174

[CR12] Bastin J, Sroussi M, Nemazanyy I, Laurent-Puig P, Mouillet-Richard S, Djouadi F. Downregulation of mitochondrial complex I induces ROS production in colorectal cancer subtypes that differently controls migration. J Transl Med. 2023;21(1):522. 10.1186/s12967-023-04341-x. 37533102 10.1186/s12967-023-04341-x PMC10398918

[CR13] Bishnu A, Sakpal A, Ghosh N, Choudhury P, Chaudhury K, Ray P. Long term treatment of metformin impedes development of chemoresistance by regulating cancer stem cell differentiation through taurine generation in ovarian cancer cells. Int J Biochem Cell Biol. 2019;107:116–27. 10.1016/j.biocel.2018.12.016. 30593952 10.1016/j.biocel.2018.12.016

[CR14] Bourges I, Ramus C, Mousson de Camaret B, et al. Structural organization of mitochondrial human complex I: role of the ND4 and ND5 mitochondria-encoded subunits and interaction with prohibitin. Biochem J. 2004;383(Pt3):491–9. 10.1042/bj20040256. 15250827 10.1042/bj20040256 PMC1133742

[CR15] Bricker DK, Taylor EB, Schell JC, et al. A mitochondrial pyruvate carrier required for pyruvate uptake in yeast, drosophila, and humans. Science. 2012;337(6090):96–100. 10.1126/science.1218099. 22628558 10.1126/science.1218099 PMC3690818

[CR16] Brown MD, Torroni A, Reckord CL, Wallace DC. Phylogenetic analysis of Leber’s hereditary optic neuropathy mitochondrial DNA’s indicates multiple independent occurrences of the common mutations. Hum Mutat. 1995;6(4):311–25. 10.1002/humu.1380060405. 8680405 10.1002/humu.1380060405

[CR17] Burrage LC, Tang S, Wang J, et al. Mitochondrial myopathy, lactic acidosis, and sideroblastic anemia (MLASA) plus associated with a novel de novo mutation (m.8969G>A) in the mitochondrial encoded ATP6 gene. Mol Genet Metab. 2014;113(3):207–12. 10.1016/j.ymgme.2014.06.004. 25037980 10.1016/j.ymgme.2014.06.004 PMC4253070

[CR18] Burris HA 3rd. Dual kinase inhibition in the treatment of breast cancer: initial experience with the EGFR/ErbB-2 inhibitor lapatinib. Oncologist. 2004;9(Suppl 3):10–5. 10.1634/theoncologist.9-suppl_3-10. 15163842 10.1634/theoncologist.9-suppl_3-10

[CR19] Bushel PR, Ward J, Burkholder A, Li J, Anchang B. Mitochondrial-nuclear epistasis underlying phenotypic variation in breast cancer pathology. Sci Rep. 2022;12(1):1393. 10.1038/s41598-022-05148-4. 35082309 10.1038/s41598-022-05148-4 PMC8791930

[CR20] Bussard KM, Siracusa LD. Understanding mitochondrial polymorphisms in cancer. Cancer Res. 2017;77(22):6051–9. 10.1158/0008-5472.Can-17-1939. 29097610 10.1158/0008-5472.Can-17-1939

[CR21] Carrer A, Trefely S, Zhao S, et al. Acetyl-CoA metabolism supports multistep pancreatic tumorigenesis. Cancer Discov. 2019;9(3):416–35. 10.1158/2159-8290.Cd-18-0567. 30626590 10.1158/2159-8290.Cd-18-0567 PMC6643997

[CR22] Castagna AE, Addis J, McInnes RR, et al. Late onset leigh syndrome and ataxia due to a T to C mutation at bp 9185 of mitochondrial DNA. Am J Med Genet A. 2007;143(8):808–16. 10.1002/ajmg.a.31637. 10.1002/ajmg.a.31637 17352390

[CR23] Cavalcante GC, Marinho ANR, Anaissi AK, et al. Whole mitochondrial genome sequencing highlights mitochondrial impact in gastric cancer. Sci Rep. 2019;9(1):15716. 10.1038/s41598-019-51951-x. 31673122 10.1038/s41598-019-51951-x PMC6823544

[CR24] Chae YC, Kim JH. Cancer stem cell metabolism: target for cancer therapy. BMB Rep. 2018;51(7):319–26. 10.5483/bmbrep.2018.51.7.112. 29764565 10.5483/bmbrep.2018.51.7.112 PMC6089865

[CR25] Chen H, Chan DC. Mitochondrial dynamics–fusion, fission, movement, and mitophagy–in neurodegenerative diseases. Hum Mol Genet. 2009;18(R2):R169–76. 10.1093/hmg/ddp326. 19808793 10.1093/hmg/ddp326 PMC2758711

[CR26] Chen W, Zou P, Zhao Z, et al. Synergistic antitumor activity of rapamycin and EF24 via increasing ROS for the treatment of gastric cancer. Redox Biol. 2016;10:78–89. 10.1016/j.redox.2016.09.006. 27697670 10.1016/j.redox.2016.09.006 PMC5048112

[CR27] Chen L, Zhou J, Zhao Z, et al. Low expression of phosphodiesterase 2 (PDE2A) promotes the progression by regulating mitochondrial morphology and ATP content and predicts poor prognosis in hepatocellular carcinoma. Cells. 2022. 10.3390/cells12010068. 36611861 10.3390/cells12010068 PMC9818237

[CR28] Chen B, Lei S, Yin X, et al. Mitochondrial respiration inhibition suppresses papillary thyroid carcinoma via PI3K/Akt/FoxO1/Cyclin D1 pathway. Front Oncol. 2022;12: 900444. 10.3389/fonc.2022.900444. 35865479 10.3389/fonc.2022.900444 PMC9295996

[CR29] Cheng L, Yang S, Yang Y, et al. Global gene expression and functional network analysis of gastric cancer identify extended pathway maps and GPRC5A as a potential biomarker. Cancer Lett. 2012;326(1):105–13. 10.1016/j.canlet.2012.07.031. 22867946 10.1016/j.canlet.2012.07.031

[CR30] Choudhury AR, Singh KK. Mitochondrial determinants of cancer health disparities. Semin Cancer Biol. 2017;47:125–46. 10.1016/j.semcancer.2017.05.001. 28487205 10.1016/j.semcancer.2017.05.001 PMC5673596

[CR31] Corbet C, Bastien E, Draoui N, et al. Interruption of lactate uptake by inhibiting mitochondrial pyruvate transport unravels direct antitumor and radiosensitizing effects. Nat Commun. 2018;9(1):1208. 10.1038/s41467-018-03525-0. 29572438 10.1038/s41467-018-03525-0 PMC5865202

[CR32] Craig K, Elliott HR, Keers SM, et al. Episodic ataxia and hemiplegia caused by the 8993T≥C mitochondrial DNA mutation. J Med Genet. 2007;44(12):797–9. 10.1136/jmg.2007.052902. 18055910 10.1136/jmg.2007.052902 PMC2652821

[CR33] Dasgupta S, Soudry E, Mukhopadhyay N, et al. Mitochondrial DNA mutations in respiratory complex-I in never-smoker lung cancer patients contribute to lung cancer progression and associated with EGFR gene mutation. J Cell Physiol. 2012;227(6):2451–60. 10.1002/jcp.22980. 21830212 10.1002/jcp.22980 PMC3256258

[CR34] de Vries DD, van Engelen BG, Gabreëls FJ, Ruitenbeek W, van Oost BA. A second missense mutation in the mitochondrial ATPase 6 gene in leigh’s syndrome. Ann Neurol. 1993;34(3):410–2. 10.1002/ana.410340319. 8395787 10.1002/ana.410340319

[CR35] De Vries DD, Went LN, Bruyn GW, et al. Genetic and biochemical impairment of mitochondrial complex I activity in a family with Leber hereditary optic neuropathy and hereditary spastic dystonia. Am J Hum Genet. 1996;58(4):703–11. 8644732 PMC1914692

[CR36] DeBalsi KL, Hoff KE, Copeland WC. Role of the mitochondrial DNA replication machinery in mitochondrial DNA mutagenesis, aging and age-related diseases. Ageing Res Rev. 2017;33:89–104. 10.1016/j.arr.2016.04.006. 27143693 10.1016/j.arr.2016.04.006 PMC5086445

[CR37] Dekkers JF, Whittle JR, Vaillant F, et al. Modeling breast cancer using CRISPR-Cas9-mediated engineering of human breast organoids. J Natl Cancer Inst. 2020;112(5):540–4. 10.1093/jnci/djz196. 31589320 10.1093/jnci/djz196 PMC7225674

[CR38] Deschênes-Simard X, Rowell MC, Ferbeyre G. Metformin turns off the metabolic switch of pancreatic cancer. Aging. 2019;11(23):10793–5. 10.18632/aging.102622. 31831715 10.18632/aging.102622 PMC6932906

[CR39] Dewangan J, Srivastava S, Rath SK. Salinomycin: a new paradigm in cancer therapy. Tumour Biol. 2017;39(3):1010428317695035. 10.1177/1010428317695035. 28349817 10.1177/1010428317695035

[CR40] Diehn M, Cho RW, Lobo NA, et al. Association of reactive oxygen species levels and radioresistance in cancer stem cells. Nature. 2009;458(7239):780–3. 10.1038/nature07733. 19194462 10.1038/nature07733 PMC2778612

[CR41] Dou X, Fu Q, Long Q, et al. PDK4-dependent hypercatabolism and lactate production of senescent cells promotes cancer malignancy. Nat Metab. 2023;5(11):1887–910. 10.1038/s42255-023-00912-w. 37903887 10.1038/s42255-023-00912-w PMC10663165

[CR42] Fonseca Cabral G, Dos Santos A, Pinheiro J, Vidal AF, Santos S, Ribeiro-Dos-Santos Â. piRNAs in gastric cancer: a new approach towards translational research. Int J Mol Sci. 2020. 10.3390/ijms21062126. 32204558 10.3390/ijms21062126 PMC7139476

[CR43] Frezza C. Mitochondrial metabolites: undercover signalling molecules. Interfac Focus. 2017;7(2):20160100. 10.1098/rsfs.2016.0100. 10.1098/rsfs.2016.0100 PMC5311903 28382199

[CR44] Frezza C, Gottlieb E. Mitochondria in cancer: not just innocent bystanders. Semin Cancer Biol. 2009;19(1):4–11. 10.1016/j.semcancer.2008.11.008. 19101633 10.1016/j.semcancer.2008.11.008

[CR45] Ga T, Wt X, Xl Z, et al. CCBE1 promotes mitochondrial fusion by inhibiting the TGFβ-DRP1 axis to prevent the progression of hepatocellular carcinoma. Matrix Biol J Int Soc Matrix Biol. 2023;117:31–45. 10.1016/j.matbio.2023.02.007. 10.1016/j.matbio.2023.02.007 36849082

[CR46] García Rubiño ME, Carrillo E, Ruiz Alcalá G, Domínguez-Martín A, J AM, Boulaiz H. Phenformin as an anticancer agent: challenges and prospects. Int J Mol Sci. 2019. 10.3390/ijms20133316. 31284513 10.3390/ijms20133316 PMC6651400

[CR47] Gibellini L, Losi L, De Biasi S, et al. LonP1 differently modulates mitochondrial function and bioenergetics of primary versus metastatic colon cancer cells. Front Oncol. 2018;8:254. 10.3389/fonc.2018.00254. 30038898 10.3389/fonc.2018.00254 PMC6046640

[CR48] Gogvadze V, Orrenius S, Zhivotovsky B. Mitochondria in cancer cells: what is so special about them? Trend Cell Biol. 2008;18(4):165–73. 10.1016/j.tcb.2008.01.006 18296052

[CR49] Grigorian M, Ambartsumian N, Lykkesfeldt AE, et al. Effect of mts1 (S100A4) expression on the progression of human breast cancer cells. Int J Cancer. 1996;67(6):831–41. 10.1002/(sici)1097-0215(19960917)67:6%3c831::Aid-ijc13%3e3.0.Co;2-4. 8824556 10.1002/(sici)1097-0215(19960917)67:6<831::Aid-ijc13>3.0.Co;2-4

[CR50] Grzybowska-Szatkowska L, Slaska B, Rzymowska J, Brzozowska A, Floriańczyk B. Novel mitochondrial mutations in the ATP6 and ATP8 genes in patients with breast cancer. Mol Med Rep. 2014;10(4):1772–8. 10.3892/mmr.2014.2471. 25110199 10.3892/mmr.2014.2471 PMC4148381

[CR51] Guantes R, Rastrojo A, Neves R, Lima A, Aguado B, Iborra FJ. Global variability in gene expression and alternative splicing is modulated by mitochondrial content. Genome Res. 2015;25(5):633–44. 10.1101/gr.178426.114. 25800673 10.1101/gr.178426.114 PMC4417112

[CR52] Guaragnella N, Giannattasio S, Moro L. Mitochondrial dysfunction in cancer chemoresistance. Biochem Pharmacol. 2014;92(1):62–72. 10.1016/j.bcp.2014.07.027. 25107705 10.1016/j.bcp.2014.07.027

[CR53] Guerra F, Guaragnella N, Arbini AA, Bucci C, Giannattasio S, Moro L. Mitochondrial dysfunction: a novel potential driver of Epithelial-to-mesenchymal transition in cancer. Front Oncol. 2017;7:295. 10.3389/fonc.2017.00295. 29250487 10.3389/fonc.2017.00295 PMC5716985

[CR54] Guo R, Zong S, Wu M, Gu J, Yang M. Architecture of human mitochondrial respiratory megacomplex I(2)III(2)IV(2). Cell. 2017;170(6):1247-1257.e12. 10.1016/j.cell.2017.07.050. 28844695 10.1016/j.cell.2017.07.050

[CR55] He SJ, Cheng J, Feng X, Yu Y, Tian L, Huang Q. The dual role and therapeutic potential of high-mobility group box 1 in cancer. Oncotarget. 2017;8(38):64534–50. 10.18632/oncotarget.17885. 28969092 10.18632/oncotarget.17885 PMC5610024

[CR56] Hermawan A, Wagner E, Roidl A. Consecutive salinomycin treatment reduces doxorubicin resistance of breast tumor cells by diminishing drug efflux pump expression and activity. Oncol Rep. 2016;35(3):1732–40. 10.3892/or.2015.4509. 26708059 10.3892/or.2015.4509

[CR57] Herzig S, Raemy E, Montessuit S, et al. Identification and functional expression of the mitochondrial pyruvate carrier. Science. 2012;337(6090):93–6. 10.1126/science.1218530. 22628554 10.1126/science.1218530

[CR58] Hofhaus G, Attardi G. Lack of assembly of mitochondrial DNA-encoded subunits of respiratory NADH dehydrogenase and loss of enzyme activity in a human cell mutant lacking the mitochondrial ND4 gene product. Embo j. 1993;12(8):3043–8. 10.1002/j.1460-2075.1993.tb05973.x. 8344246 10.1002/j.1460-2075.1993.tb05973.x PMC413569

[CR59] Holt IJ, Harding AE, Petty RK, Morgan-Hughes JA. A new mitochondrial disease associated with mitochondrial DNA heteroplasmy. Am J Hum Genet. 1990;46(3):428–33. 2137962 PMC1683641

[CR60] Hong M, Tao S, Zhang L, et al. RNA sequencing: new technologies and applications in cancer research. J Hematol Oncol. 2020;13(1):166. 10.1186/s13045-020-01005-x. 33276803 10.1186/s13045-020-01005-x PMC7716291

[CR61] Hong J, Shiba-Ishii A, Kim Y, Noguchi M, Sakamoto N. Ovarian carcinoma immunoreactive antigen domain 2 controls mitochondrial apoptosis in lung adenocarcinoma. Cancer Sci. 2021;112(12):5114–26. 10.1111/cas.15160. 34628698 10.1111/cas.15160 PMC8645747

[CR62] Huang Q, Zhan L, Cao H, et al. Increased mitochondrial fission promotes autophagy and hepatocellular carcinoma cell survival through the ROS-modulated coordinated regulation of the NFKB and TP53 pathways. Autophagy. 2016;12(6):999–1014. 10.1080/15548627.2016.1166318. 27124102 10.1080/15548627.2016.1166318 PMC4922447

[CR63] Huang CY, Chiang SF, Chen WT, et al. HMGB1 promotes ERK-mediated mitochondrial Drp1 phosphorylation for chemoresistance through RAGE in colorectal cancer. Cell Death Dis. 2018;9(10):1004. 10.1038/s41419-018-1019-6. 30258050 10.1038/s41419-018-1019-6 PMC6158296

[CR64] Huang L, Wei B, Zhao Y, Gong X, Chen L. DYNLT1 promotes mitochondrial metabolism to fuel breast cancer development by inhibiting ubiquitination degradation of VDAC1. Mol Med. 2023;29(1):72. 10.1186/s10020-023-00663-0. 37280526 10.1186/s10020-023-00663-0 PMC10245490

[CR65] Ippolito L, Morandi A, Taddei ML, et al. Cancer-associated fibroblasts promote prostate cancer malignancy via metabolic rewiring and mitochondrial transfer. Oncogene. 2019;38(27):5339–55. 10.1038/s41388-019-0805-7. 30936458 10.1038/s41388-019-0805-7

[CR66] Jangamreddy JR, Jain MV, Hallbeck AL, Roberg K, Lotfi K, Łos MJ. Glucose starvation-mediated inhibition of salinomycin induced autophagy amplifies cancer cell specific cell death. Oncotarget. 2015;6(12):10134–45. 10.18632/oncotarget.3548. 25912307 10.18632/oncotarget.3548 PMC4496345

[CR67] Jara JA, Castro-Castillo V, Saavedra-Olavarría J, et al. Antiproliferative and uncoupling effects of delocalized, lipophilic, cationic gallic acid derivatives on cancer cell lines. Validation in vivo in singenic mice. J Med Chem. 2014;57(6):2440–54. 10.1021/jm500174v. 24568614 10.1021/jm500174v

[CR68] Jayasekera LP, Ranasinghe R, Senathilake KS, et al. Mitochondrial genome in sporadic breast cancer: a case control study and a proteomic analysis in a Sinhalese cohort from Sri Lanka. PLoS ONE. 2023;18(2): e0281620. 10.1371/journal.pone.0281620. 36758048 10.1371/journal.pone.0281620 PMC9910733

[CR69] Jiang J, Li H, Qaed E, et al. Salinomycin, as an autophagy modulator– a new avenue to anticancer: a review. J Exp Clin Cancer Res. 2018;37(1):26. 10.1186/s13046-018-0680-z. 29433536 10.1186/s13046-018-0680-z PMC5809980

[CR70] Johns DR, Neufeld MJ. Cytochrome c oxidase mutations in Leber hereditary optic neuropathy. Biochem Biophys Res Commun. 1993;196(2):810–5. 10.1006/bbrc.1993.2321. 8240356 10.1006/bbrc.1993.2321

[CR71] Kalyanaraman B, Cheng G, Hardy M, et al. A review of the basics of mitochondrial bioenergetics, metabolism, and related signaling pathways in cancer cells: therapeutic targeting of tumor mitochondria with lipophilic cationic compounds. Redox Biol. 2018;14:316–27. 10.1016/j.redox.2017.09.020. 29017115 10.1016/j.redox.2017.09.020 PMC5633086

[CR72] Keightley JA, Hoffbuhr KC, Burton MD, et al. A microdeletion in cytochrome c oxidase (COX) subunit III associated with COX deficiency and recurrent myoglobinuria. Nat Genet. 1996;12(4):410–6. 10.1038/ng0496-410. 8630495 10.1038/ng0496-410

[CR73] Ko JC, Zheng HY, Chen WC, et al. Salinomycin enhances cisplatin-induced cytotoxicity in human lung cancer cells via down-regulation of AKT-dependent thymidylate synthase expression. Biochem Pharmacol. 2016;122:90–8. 10.1016/j.bcp.2016.09.022. 27666600 10.1016/j.bcp.2016.09.022

[CR74] Lai RK, Xu IM, Chiu DK, et al. NDUFA4L2 fine-tunes oxidative stress in hepatocellular carcinoma. Clin Cancer Res. 2016;22(12):3105–17. 10.1158/1078-0432.Ccr-15-1987. 26819450 10.1158/1078-0432.Ccr-15-1987

[CR75] Lamb R, Ozsvari B, Lisanti CL, et al. Antibiotics that target mitochondria effectively eradicate cancer stem cells, across multiple tumor types: treating cancer like an infectious disease. Oncotarget. 2015;6(7):4569–84. 10.18632/oncotarget.3174. 25625193 10.18632/oncotarget.3174 PMC4467100

[CR76] Lei L, Chen C, Zhao J, et al. Targeted expression of miR-7 operated by TTF-1 promoter inhibited the growth of human lung cancer through the NDUFA4 pathway. Mol Ther Nucleic Acids. 2017;6:183–97. 10.1016/j.omtn.2016.12.005. 28325285 10.1016/j.omtn.2016.12.005 PMC5363496

[CR77] Li Y, Beckman KB, Caberto C, et al. Association of genes, pathways, and haplogroups of the mitochondrial genome with the risk of colorectal cancer: the multiethnic cohort. PLoS ONE. 2015;10(9): e0136796. 10.1371/journal.pone.0136796. 26340450 10.1371/journal.pone.0136796 PMC4560485

[CR78] Li X, Han G, Li X, et al. Mitochondrial pyruvate carrier function determines cell stemness and metabolic reprogramming in cancer cells. Oncotarget. 2017;8(28):46363–80. 10.18632/oncotarget.18199. 28624784 10.18632/oncotarget.18199 PMC5542273

[CR79] Li YL, Chen CH, Chen JY, et al. Single-cell analysis reveals immune modulation and metabolic switch in tumor-draining lymph nodes. Oncoimmunology. 2020;9(1):1830513. 10.1080/2162402x.2020.1830513. 33117603 10.1080/2162402x.2020.1830513 PMC7575008

[CR80] Li X, Wang M, Li S, et al. HIF-1-induced mitochondrial ribosome protein L52: a mechanism for breast cancer cellular adaptation and metastatic initiation in response to hypoxia. Theranostics. 2021;11(15):7337–59. 10.7150/thno.57804. 34158854 10.7150/thno.57804 PMC8210597

[CR81] Li L, Li Y, Huang Y, et al. Long non-coding RNA MIF-AS1 promotes gastric cancer cell proliferation and reduces apoptosis to upregulate NDUFA4. Cancer Sci. 2018;109(12):3714–25. 10.1111/cas.13801. 30238562 10.1111/cas.13801 PMC6272088

[CR82] Lim SC, Hroudová J, Van Bergen NJ, Lopez Sanchez MI, Trounce IA, McKenzie M. Loss of mitochondrial DNA-encoded protein ND1 results in disruption of complex I biogenesis during early stages of assembly. Faseb j. 2016;30(6):2236–48. 10.1096/fj.201500137R. 26929434 10.1096/fj.201500137R

[CR83] Lin CS, Liu LT, Ou LH, Pan SC, Lin CI, Wei YH. Role of mitochondrial function in the invasiveness of human colon cancer cells. Oncol Rep. 2018;39(1):316–30. 10.3892/or.2017.6087. 29138850 10.3892/or.2017.6087

[CR84] Liolitsa D, Rahman S, Benton S, Carr LJ, Hanna MG. Is the mitochondrial complex I ND5 gene a hot-spot for MELAS causing mutations? Ann Neurol. 2003;53(1):128–32. 10.1002/ana.10435. 12509858 10.1002/ana.10435

[CR85] Liu S. The role of NDUFA4 in the proliferation of human colorectal carcinoma cells and its probable mechanism. Zunyi: Zunyi Medical University; 2020.

[CR86] Liu L, Qi L, Knifley T, et al. S100A4 alters metabolism and promotes invasion of lung cancer cells by up-regulating mitochondrial complex I protein NDUFS2. J Biol Chem. 2019;294(18):7516–27. 10.1074/jbc.RA118.004365. 30885944 10.1074/jbc.RA118.004365 PMC6509482

[CR87] Liu WL, Li CY, Cheng WC, et al. High mobility group box 1 promotes lung cancer cell migration and motility via regulation of dynamin-related protein 1. Int J Mol Sci. 2021;22(7):3628. 10.3390/ijms22073628. 33807275 10.3390/ijms22073628 PMC8036886

[CR88] Liu Z, Shi M, Ren Y, et al. Recent advances and applications of CRISPR-Cas9 in cancer immunotherapy. Mol Cancer. 2023;22(1):35. 10.1186/s12943-023-01738-6. 36797756 10.1186/s12943-023-01738-6 PMC9933290

[CR89] S Liu, D Tao, L Lei, et al. Role of type I coenzyme dehydrogenase 1α. subcomplex 4 overexpression on epithelial-mesenchymal transition in human colorectal cancer cells. J Med Postgrad. 32(06): 596–601.

[CR90] Lopes Costa A, Le Bachelier C, Mathieu L, et al. Beneficial effects of resveratrol on respiratory chain defects in patients’ fibroblasts involve estrogen receptor and estrogen-related receptor alpha signaling. Hum Mol Genet. 2014;23(8):2106–19. 10.1093/hmg/ddt603. 24365713 10.1093/hmg/ddt603

[CR91] Lu J, Chen M, Gao S, Yuan J, Zhu Z, Zou X. LY294002 inhibits the Warburg effect in gastric cancer cells by downregulating pyruvate kinase M2. Oncol Lett. 2018;15(4):4358–64. 10.3892/ol.2018.7843. 29541204 10.3892/ol.2018.7843 PMC5835956

[CR92] Lu X, Liu QX, Zhang J, et al. PINK1 overexpression promotes cell migration and proliferation via regulation of autophagy and predicts a poor prognosis in lung cancer cases. Cancer Manag Res. 2020;12:7703–14. 10.2147/cmar.S262466. 32904694 10.2147/cmar.S262466 PMC7457709

[CR93] Lu Y, Li K, Gao Y, Liang W, Wang X, Chen L. CircRNAs in gastric cancer: current research and potential clinical implications. FEBS Lett. 2021;595(21):2644–54. 10.1002/1873-3468.14196. 34561854 10.1002/1873-3468.14196

[CR94] Lucioli S, Hoffmeier K, Carrozzo R, Tessa A, Ludwig B, Santorelli FM. Introducing a novel human mtDNA mutation into the Paracoccus denitrificans COX I gene explains functional deficits in a patient. Neurogenetics. 2006;7(1):51–7. 10.1007/s10048-005-0015-z. 16284789 10.1007/s10048-005-0015-z

[CR95] Luengo A, Sullivan LB, Heiden MG. Understanding the complex-I-ty of metformin action: limiting mitochondrial respiration to improve cancer therapy. BMC Biol. 2014;12:82. 10.1186/s12915-014-0082-4. 25347702 10.1186/s12915-014-0082-4 PMC4207883

[CR96] Magrath JW, Raney WR, Kim Y. In vitro demonstration of salinomycin as a novel chemotherapeutic agent for the treatment of SOX2-positive glioblastoma cancer stem cells. Oncol Rep. 2020;44(2):777–85. 10.3892/or.2020.7642. 32627023 10.3892/or.2020.7642

[CR97] Mahmood M, Liu EM, Shergold AL, et al. Mitochondrial DNA mutations drive aerobic glycolysis to enhance checkpoint blockade response in melanoma. Nat Cancer. 2024;5(4):659–72. 10.1038/s43018-023-00721-w. 38286828 10.1038/s43018-023-00721-w PMC11056318

[CR98] Majander A, Huoponen K, Savontaus ML, Nikoskelainen E, Wikström M. Electron transfer properties of NADH:ubiquinone reductase in the ND1/3460 and the ND4/11778 mutations of the Leber hereditary optic neuroretinopathy (LHON). FEBS Lett. 1991;292(1–2):289–92. 10.1016/0014-5793(91)80886-8. 1959619 10.1016/0014-5793(91)80886-8

[CR99] Markowska A, Sajdak S, Huczyński A, Rehlis S, Markowska J. Ovarian cancer stem cells: a target for oncological therapy. Adv Clin Exp Med. 2018;27(7):1017–20. 10.17219/acem/73999. 29938937 10.17219/acem/73999

[CR100] Martínez-Ramírez M, Coral-Vázquez RM, Tenorio A, et al. Complete sequence of the ATP6 and ND3 mitochondrial genes in breast cancer tissue of postmenopausal women with different body mass indexes. Ann Diagn Pathol. 2018;32:23–7. 10.1016/j.anndiagpath.2017.09.001. 29414393 10.1016/j.anndiagpath.2017.09.001

[CR101] McCommis KS, Hodges WT, Bricker DK, et al. An ancestral role for the mitochondrial pyruvate carrier in glucose-stimulated insulin secretion. Mol Metab. 2016;5(8):602–14. 10.1016/j.molmet.2016.06.016. 27656398 10.1016/j.molmet.2016.06.016 PMC5021712

[CR102] Meng F, Wu L, Dong L, et al. EGFL9 promotes breast cancer metastasis by inducing cMET activation and metabolic reprogramming. Nat Commun. 2019;10(1):5033. 10.1038/s41467-019-13034-3. 31695034 10.1038/s41467-019-13034-3 PMC6834558

[CR103] Mi Y, Li Q, Liu B, et al. Ubiquitous mitochondrial creatine kinase promotes the progression of gastric cancer through a JNK-MAPK/JUN/HK2 axis regulated glycolysis. Gastric Cancer. 2023;26(1):69–81. 10.1007/s10120-022-01340-7. 36114400 10.1007/s10120-022-01340-7 PMC9813075

[CR104] Miller DK, Menezes MJ, Simons C, et al. Rapid identification of a novel complex I MT-ND3 m.10134C>A mutation in a Leigh syndrome patient. PLoS One. 2014;9(8): e104879. 10.1371/journal.pone.0104879. 25118196 10.1371/journal.pone.0104879 PMC4130626

[CR105] Morgenstern M, Stiller SB, Lübbert P, et al. Definition of a high-confidence mitochondrial proteome at quantitative scale. Cell Rep. 2017;19(13):2836–52. 10.1016/j.celrep.2017.06.014. 28658629 10.1016/j.celrep.2017.06.014 PMC5494306

[CR106] Morgenstern M, Peikert CD, Lübbert P, et al. Quantitative high-confidence human mitochondrial proteome and its dynamics in cellular context. Cell Metab. 2021;33(12):2464-2483.e18. 10.1016/j.cmet.2021.11.001. 34800366 10.1016/j.cmet.2021.11.001 PMC8664129

[CR107] Mottaghi-Dastjerdi N, Ghorbani A, Montazeri H, Guzzi PH. A systems biology approach to pathogenesis of gastric cancer: gene network modeling and pathway analysis. BMC Gastroenterol. 2023;23(1):248. 10.1186/s12876-023-02891-4. 37482618 10.1186/s12876-023-02891-4 PMC10364406

[CR108] Naujokat C, Steinhart R. Salinomycin as a drug for targeting human cancer stem cells. J Biomed Biotechnol. 2012;2012: 950658. 10.1155/2012/950658. 23251084 10.1155/2012/950658 PMC3516046

[CR109] Nayak AP, Kapur A, Barroilhet L, Patankar MS. Oxidative phosphorylation: a target for novel therapeutic strategies against ovarian cancer. Cancers. 2018. 10.3390/cancers10090337. 30231564 10.3390/cancers10090337 PMC6162441

[CR110] Nicholls TJ, Minczuk M. In D-loop: 40 years of mitochondrial 7S DNA. Exp Gerontol. 2014;56:175–81. 10.1016/j.exger.2014.03.027. 24709344 10.1016/j.exger.2014.03.027

[CR111] Nunnari J, Suomalainen A. Mitochondria: in sickness and in health. Cell. 2012;148(6):1145–59. 10.1016/j.cell.2012.02.035. 22424226 10.1016/j.cell.2012.02.035 PMC5381524

[CR112] Papadaki V, Erpapazoglou Z, Kokkori M, et al. IQGAP1 mediates the communication between the nucleus and the mitochondria via NDUFS4 alternative splicing. NAR Cancer. 2023. 10.1093/narcan/zcad046. 37636315 10.1093/narcan/zcad046 PMC10448856

[CR113] Park SH, Lee AR, Choi K, Joung S, Yoon JB, Kim S. TOMM20 as a potential therapeutic target of colorectal cancer. BMB Rep. 2019;52(12):712–7. 31818360 10.5483/BMBRep.2019.52.12.249 PMC6941759

[CR114] Pfanner N, Warscheid B, Wiedemann N. Mitochondrial proteins: from biogenesis to functional networks. Nat Rev Mol Cell Biol. 2019;20(5):267–84. 10.1038/s41580-018-0092-0. 30626975 10.1038/s41580-018-0092-0 PMC6684368

[CR115] Philley JV, Kannan A, Qin W, et al. Complex-I alteration and enhanced mitochondrial fusion are associated with prostate cancer progression. J Cell Physiol. 2016;231(6):1364–74. 10.1002/jcp.25240. 26530043 10.1002/jcp.25240 PMC5741292

[CR116] Poewe W, Seppi K, Tanner CM, et al. Parkinson disease. Nat Rev Dis Primer. 2017;3:17013. 10.1038/nrdp.2017.13. 10.1038/nrdp.2017.13 28332488

[CR117] Power MD, Kiefer MC, Barr PJ, Reeves R. Nucleotide sequence of human mitochondrial cytochrome c oxidase II cDNA. Nucl Acid Res. 1989;17(16):6734. 10.1093/nar/17.16.6734. 10.1093/nar/17.16.6734 PMC318375 2550900

[CR118] Qi D, Liu Y, Li J, Huang JH, Hu X, Wu E. Salinomycin as a potent anticancer stem cell agent: State of the art and future directions. Med Res Rev. 2022;42(3):1037–63. 10.1002/med.21870. 34786735 10.1002/med.21870 PMC9298915

[CR119] Rahman S, Taanman JW, Cooper JM, et al. A missense mutation of cytochrome oxidase subunit II causes defective assembly and myopathy. Am J Hum Genet. 1999;65(4):1030–9. 10.1086/302590. 10486321 10.1086/302590 PMC1288235

[CR120] Rantamäki MT, Soini HK, Finnilä SM, Majamaa K, Udd B. Adult-onset ataxia and polyneuropathy caused by mitochondrial 8993T≥C mutation. Ann Neurol. 2005;58(2):337–40. 10.1002/ana.20555. 16049925 10.1002/ana.20555

[CR121] Rath S, Sharma R, Gupta R, et al. MitoCarta3.0: an updated mitochondrial proteome now with sub-organelle localization and pathway annotations. Nucl Acid Res. 2021;49(D1):D1541-d1547. 10.1093/nar/gkaa1011. 10.1093/nar/gkaa1011 PMC7778944 33174596

[CR122] Reznik E, Miller ML, Şenbabaoğlu Y, et al. Mitochondrial DNA copy number variation across human cancers. Elife. 2016. 10.7554/eLife.10769. 26901439 10.7554/eLife.10769 PMC4775221

[CR123] Sasaki Y, Takagane K, Konno T, et al. Expression of asporin reprograms cancer cells to acquire resistance to oxidative stress. Cancer Sci. 2021;112(3):1251–61. 10.1111/cas.14794. 33393151 10.1111/cas.14794 PMC7935789

[CR124] Schulz C, Schendzielorz A, Rehling P. Unlocking the presequence import pathway. Trend Cell Biol. 2015;25(5):265–75. 10.1016/j.tcb.2014.12.001. 10.1016/j.tcb.2014.12.001 25542066

[CR125] Shao N, Qiu H, Liu J, Xiao D, Zhao J, Chen C, Wan J, Guo M, Liang G, Zhao X, Xu L. Targeting lipid metabolism of macrophages: a new strategy for tumor therapy. J Adv Res. 2024;S2090–1232(24):00071–7. 10.1016/j.jare.2024.02.009.10.1016/j.jare.2024.02.00938373649

[CR126] Shaughnessy DT, McAllister K, Worth L, et al. Mitochondria, energetics, epigenetics, and cellular responses to stress. Environ Health Perspect. 2014;122(12):1271–8. 10.1289/ehp.1408418. 25127496 10.1289/ehp.1408418 PMC4256704

[CR127] Shiraishi T, Verdone JE, Huang J, et al. Glycolysis is the primary bioenergetic pathway for cell motility and cytoskeletal remodeling in human prostate and breast cancer cells. Oncotarget. 2015;6(1):130–43. 10.18632/oncotarget.2766. 25426557 10.18632/oncotarget.2766 PMC4381583

[CR128] Siegel RL, Miller KD, Goding Sauer A, et al. (2019) Colorectal cancer statistics, 2020. CA Cancer J Clin. 2020;70(3):145–64. 10.3322/caac.21601. 32133645 10.3322/caac.21601

[CR129] Singh K, Poteryakhina A, Zheltukhin A, et al. NLRX1 acts as tumor suppressor by regulating TNF-α induced apoptosis and metabolism in cancer cells. Biochimica Et Biophys Acta BBA Mol Cell Res. 2015;1853(5):1073–86. 10.1016/j.bbamcr.2015.01.016. 10.1016/j.bbamcr.2015.01.016 25639646

[CR130] Singh K, Roy M, Prajapati P, et al. NLRX1 regulates TNF-α-induced mitochondria-lysosomal crosstalk to maintain the invasive and metastatic potential of breast cancer cells. Biochimica Et Biophysica Acta BBA Mol Basis Dis. 2019;1865(6):1460–76. 10.1016/j.bbadis.2019.02.018. 10.1016/j.bbadis.2019.02.018 30802640

[CR131] Song L, Rawal B, Nemeth JA, Haura EB. JAK1 activates STAT3 activity in non-small-cell lung cancer cells and IL-6 neutralizing antibodies can suppress JAK1-STAT3 signaling. Mol Cancer Ther. 2011;10(3):481–94. 10.1158/1535-7163.Mct-10-0502. 21216930 10.1158/1535-7163.Mct-10-0502 PMC4084653

[CR132] Stockwell BR. Ferroptosis turns 10: Emerging mechanisms, physiological functions, and therapeutic applications. Cell. 2022;185(14):2401–21. 10.1016/j.cell.2022.06.003. 35803244 10.1016/j.cell.2022.06.003 PMC9273022

[CR133] Sun W, Hu C, Wang T, et al. Glia maturation factor beta as a novel biomarker and therapeutic target for hepatocellular carcinoma. Front Oncol. 2021;11:744331. 10.3389/fonc.2021.744331. 34796110 10.3389/fonc.2021.744331 PMC8593204

[CR134] Sung AY, Floyd BJ, Pagliarini DJ. Systems biochemistry approaches to defining mitochondrial protein function. Cell Metab. 2020;31(4):669–78. 10.1016/j.cmet.2020.03.011. 32268114 10.1016/j.cmet.2020.03.011 PMC7176052

[CR135] Sung H, Ferlay J, Siegel RL, et al. Global cancer statistics 2020: GLOBOCAN estimates of incidence and mortality worldwide for 36 cancers in 185 countries. CA Cancer J Clin. 2021;71(3):209–49. 10.3322/caac.21660. 33538338 10.3322/caac.21660

[CR136] Tamura M, Matsui H, Tomita T, et al. Mitochondrial reactive oxygen species accelerate gastric cancer cell invasion. J Clin Biochem Nut. 2014;54(1):12–7. 10.3164/jcbn.13-36. 10.3164/jcbn.13-36 PMC3882482 24426185

[CR137] Tan H, Zhang S, Zhang J, et al. Long non-coding RNAs in gastric cancer: new emerging biological functions and therapeutic implications. Theranostics. 2020;10(19):8880–902. 10.7150/thno.47548. 32754285 10.7150/thno.47548 PMC7392009

[CR138] Tang Y, Li Z, Shi ZX. Mechanisms of the suppression of proliferation and invasion ability mediated by microRNA-147b in esophageal squamous cell carcinoma. Zhonghua Yi Xue Za Zhi. 2018;98(26):2092–8. 10.3760/cma.j.issn.0376-2491.2018.26.007. 30032507 10.3760/cma.j.issn.0376-2491.2018.26.007

[CR139] Tang H, Peng S, Dong Y, et al. MARCH5 overexpression contributes to tumor growth and metastasis and associates with poor survival in breast cancer. Cancer Manag Res. 2019;11:201–15. 10.2147/cmar.S190694. 30636894 10.2147/cmar.S190694 PMC6307674

[CR140] Tebbenkamp ATN, Varela L, Choi J, et al. The 7q11.23 protein DNAJC30 interacts with ATP synthase and links mitochondria to brain development. Cell. 2018;175(4):1088-1104. e23. 10.1016/j.cell.2018.09.014. 30318146 10.1016/j.cell.2018.09.014 PMC6459420

[CR141] Tello D, Balsa E, Acosta-Iborra B, et al. Induction of the mitochondrial NDUFA4L2 protein by HIF-1α decreases oxygen consumption by inhibiting complex I activity. Cell Metab. 2011;14(6):768–79. 10.1016/j.cmet.2011.10.008. 22100406 10.1016/j.cmet.2011.10.008

[CR142] Thapa S, Lalrohlui F, Ghatak S, et al. Mitochondrial complex I and V gene polymorphisms associated with breast cancer in mizo-mongloid population. Breast Cancer. 2016;23(4):607–16. 10.1007/s12282-015-0611-1. 25896597 10.1007/s12282-015-0611-1

[CR143] Thompson CB. Metabolic enzymes as oncogenes or tumor suppressors. N Engl J Med. 2009;360(8):813–5. 10.1056/NEJMe0810213. 19228626 10.1056/NEJMe0810213 PMC2848669

[CR144] Thyagarajan D, Shanske S, Vazquez-Memije M, De Vivo D, DiMauro S. A novel mitochondrial ATPase 6 point mutation in familial bilateral striatal necrosis. Ann Neurol. 1995;38(3):468–72. 10.1002/ana.410380321. 7668837 10.1002/ana.410380321

[CR145] Tripathi A, Shrinet K, Kumar A. HMGB1 protein as a novel target for cancer. Toxicol Rep. 2019;6:253–61. 10.1016/j.toxrep.2019.03.002. 30911468 10.1016/j.toxrep.2019.03.002 PMC6416660

[CR146] Triska P, Kaneva K, Merkurjev D, et al. Landscape of germline and somatic mitochondrial DNA mutations in pediatric malignancies. Cancer Res. 2019;79(7):1318–30. 10.1158/0008-5472.Can-18-2220. 30709931 10.1158/0008-5472.Can-18-2220 PMC6445760

[CR147] Trotta AP, Chipuk JE. Mitochondrial dynamics as regulators of cancer biology. Cell Mol Life Sci. 2017;74(11):1999–2017. 10.1007/s00018-016-2451-3. 28083595 10.1007/s00018-016-2451-3 PMC5419868

[CR148] Ugalde C, Triepels RH, Coenen MJ, et al. Impaired complex I assembly in a Leigh syndrome patient with a novel missense mutation in the ND6 gene. Ann Neurol. 2003;54(5):665–9. 10.1002/ana.10734. 14595656 10.1002/ana.10734

[CR149] Ugalde C, Hinttala R, Timal S, et al. Mutated ND2 impairs mitochondrial complex I assembly and leads to Leigh syndrome. Mol Genet Metab. 2007;90(1):10–4. 10.1016/j.ymgme.2006.08.003. 16996290 10.1016/j.ymgme.2006.08.003

[CR150] Varlamov DA, Kudin AP, Vielhaber S, et al. Metabolic consequences of a novel missense mutation of the mtDNA CO I gene. Hum Mol Genet. 2002;11(16):1797–805. 10.1093/hmg/11.16.1797. 12140182 10.1093/hmg/11.16.1797

[CR151] Viale A, Corti D, Draetta GF. Tumors and mitochondrial respiration: a neglected connection. Cancer Res. 2015;75(18):3685–6. 10.1158/0008-5472.Can-15-0491. 26374463 10.1158/0008-5472.Can-15-0491

[CR152] Wallace DC. Mitochondria and cancer. Nat Rev Cancer. 2012;12(10):685–98. 10.1038/nrc3365. 23001348 10.1038/nrc3365 PMC4371788

[CR153] Wallace L, Mehrabi S, Bacanamwo M, Yao X, Aikhionbare FO. Expression of mitochondrial genes MT-ND1, MT-ND6, MT-CYB, MT-COI, MT-ATP6, and 12S/MT-RNR1 in colorectal adenopolyps. Tumour Biol. 2016;37(9):12465–75. 10.1007/s13277-016-5101-3. 27333991 10.1007/s13277-016-5101-3 PMC5661973

[CR154] Wan L, Wang Y, Zhang Z, et al. Elevated TEFM expression promotes growth and metastasis through activation of ROS/ERK signaling in hepatocellular carcinoma. Cell Death Dis. 2021;12(4):325. 10.1038/s41419-021-03618-7. 33771980 10.1038/s41419-021-03618-7 PMC7997956

[CR155] Wang X, Moraes CT. Increases in mitochondrial biogenesis impair carcinogenesis at multiple levels. Mol Oncol. 2011;5(5):399–409. 10.1016/j.molonc.2011.07.008. 21855427 10.1016/j.molonc.2011.07.008 PMC3183410

[CR156] Wang YC, Chao TK, Chang CC, Yo YT, Yu MH, Lai HC. Drug screening identifies niclosamide as an inhibitor of breast cancer stem-like cells. PLoS ONE. 2013;8(9): e74538. 10.1371/journal.pone.0074538. 24058587 10.1371/journal.pone.0074538 PMC3776833

[CR157] Wang D, Yin L, Wei J, Yang Z, Jiang G. ATP citrate lyase is increased in human breast cancer, depletion of which promotes apoptosis. Tumour Biol. 2017;39(4):1010428317698338. 10.1177/1010428317698338. 28443474 10.1177/1010428317698338

[CR158] Wang H, Wu Y, Wang Z, et al. The LncRNA FEZF1-AS1 promotes tumor proliferation in colon cancer by regulating the mitochondrial protein PCK2. Oncol Res. 2021;29(3):201–15. 10.32604/or.2022.03553. 37304670 10.32604/or.2022.03553 PMC10208053

[CR159] Wang SW, Gao C, Zheng YM, et al. Current applications and future perspective of CRISPR/Cas9 gene editing in cancer. Mol Cancer. 2022;21(1):57. 10.1186/s12943-022-01518-8. 35189910 10.1186/s12943-022-01518-8 PMC8862238

[CR160] Ware SM, El-Hassan N, Kahler SG, et al. Infantile cardiomyopathy caused by a mutation in the overlapping region of mitochondrial ATPase 6 and 8 genes. J Med Genet. 2009;46(5):308–14. 10.1136/jmg.2008.063149. 19188198 10.1136/jmg.2008.063149

[CR161] Wittig I, Malacarne PF. Complexome profiling: assembly and remodeling of protein complexes. Int J Mol Sci. 2021. 10.3390/ijms22157809. 34360575 10.3390/ijms22157809 PMC8346016

[CR162] Wu J, Deng Z, Zhu Y, Dou G, Li J, Huang L. Overexpression of miR-431–5p impairs mitochondrial function and induces apoptosis in gastric cancer cells via the Bax/Bcl-2/caspase3 pathway. Nan Fang Yi Ke Da Xue Xue Bao J South Med Univ. 2023;43(4):537–43. 10.12122/j.issn.1673-4254.2023.04.05 PMC10202784 37202188

[CR163] Wu J, Deng Z, Zhu Y, Dou G, Li J, Huang L. Overexpression of miR-431-5p impairs mitochondrial function and induces apoptosis in gastric cancer cells via the Bax/Bcl-2/caspase3 pathway. Nan Fang Yi Ke Da Xue Xue Bao. 2023;43(4):537–43. 10.12122/j.issn.1673-4254.2023.04.05. 37202188 10.12122/j.issn.1673-4254.2023.04.05 PMC10202784

[CR164] Xin M, Qiao Z, Li J, et al. miR-22 inhibits tumor growth and metastasis by targeting ATP citrate lyase: evidence in osteosarcoma, prostate cancer, cervical cancer and lung cancer. Oncotarget. 2016;7(28):44252–65. 10.18632/oncotarget.10020. 27317765 10.18632/oncotarget.10020 PMC5190093

[CR165] Xu Q, Zong L, Chen X, et al. Resveratrol in the treatment of pancreatic cancer. Ann N Y Acad Sci. 2015;1348(1):10–9. 10.1111/nyas.12837. 26284849 10.1111/nyas.12837

[CR166] Xu Y, Zhou J, Yuan Q, et al. Quantitative detection of circulating MT-ND1 as a potential biomarker for colorectal cancer. Bosn J Basic Med Sci. 2021;21(5):577–86. 10.17305/bjbms.2021.5576. 33823124 10.17305/bjbms.2021.5576 PMC8381205

[CR167] Xu W, Lai Y, Pan Y, et al. m6A RNA methylation-mediated NDUFA4 promotes cell proliferation and metabolism in gastric cancer. Cell Death Dis. 2022;13(8):715. 10.1038/s41419-022-05132-w. 35977935 10.1038/s41419-022-05132-w PMC9385701

[CR168] Yang Y, Huang Y, Lin W, et al. Host miRNAs-microbiota interactions in gastric cancer. J Transl Med. 2022;20(1):52. 10.1186/s12967-022-03264-3. 35093110 10.1186/s12967-022-03264-3 PMC8800214

[CR169] Yo YT, Lin YW, Wang YC, et al. Growth inhibition of ovarian tumor-initiating cells by niclosamide. Mol Cancer Ther. 2012;11(8):1703–12. 10.1158/1535-7163.Mct-12-0002. 22576131 10.1158/1535-7163.Mct-12-0002

[CR170] Yu M, Nguyen ND, Huang Y, et al. Mitochondrial fusion exploits a therapeutic vulnerability of pancreatic cancer. JCI Insight. 2019. 10.1172/jci.insight.126915. 31335325 10.1172/jci.insight.126915 PMC6777817

[CR171] Yuan Y, Wang W, Li H, et al. Nonsense and missense mutation of mitochondrial ND6 gene promotes cell migration and invasion in human lung adenocarcinoma. BMC Cancer. 2015;15:346. 10.1186/s12885-015-1349-z. 25934296 10.1186/s12885-015-1349-z PMC4425906

[CR172] Yuan Y, Ju YS, Kim Y, et al. Comprehensive molecular characterization of mitochondrial genomes in human cancers. Nat Genet. 2020;52(3):342–52. 10.1038/s41588-019-0557-x. 32024997 10.1038/s41588-019-0557-x PMC7058535

[CR173] Zeviani M, Antozzi C. Defects of mitochondrial DNA. Brain Pathol. 1992;2(2):121–32. 10.1111/j.1750-3639.1992.tb00680.x. 1341953 10.1111/j.1750-3639.1992.tb00680.x

[CR174] Zhang X, Fryknäs M, Hernlund E, et al. Induction of mitochondrial dysfunction as a strategy for targeting tumour cells in metabolically compromised microenvironments. Nat Commun. 2014;5:3295. 10.1038/ncomms4295. 24548894 10.1038/ncomms4295 PMC3929804

[CR175] Zhang W, Zhang SL, Hu X, Tam KY. Targeting tumor metabolism for cancer treatment: is pyruvate dehydrogenase kinases (PDKs) a viable anticancer target? Int J Biol Sci. 2015;11(12):1390–400. 10.7150/ijbs.13325. 26681918 10.7150/ijbs.13325 PMC4671996

[CR176] Zhang P, Yang M, Zhang Y, et al. Dissecting the single-cell transcriptome network underlying gastric premalignant lesions and early gastric cancer. Cell Rep. 2019;27(6):1934-1947.e5. 10.1016/j.celrep.2019.04.052. 31067475 10.1016/j.celrep.2019.04.052

[CR177] Zhang Y, Ge M, Chen Y, et al. NDUFA4 promotes cell proliferation by enhancing oxidative phosphorylation in pancreatic adenocarcinoma. J Bioenerg Biomembr. 2022;54(5–6):283–91. 10.1007/s10863-022-09949-0. 36307669 10.1007/s10863-022-09949-0

[CR178] Zhang D, Man D, Lu J, et al. Mitochondrial TSPO promotes hepatocellular carcinoma progression through ferroptosis inhibition and immune evasion. Adv Sci. 2023;10(15):e2206669. 10.1002/advs.202206669. 10.1002/advs.202206669 PMC10214260 36994647

[CR179] Zhao J, Zhang J, Yu M, et al. Mitochondrial dynamics regulates migration and invasion of breast cancer cells. Oncogene. 2013;32(40):4814–24. 10.1038/onc.2012.494. 23128392 10.1038/onc.2012.494 PMC3911914

[CR180] Zhao H, Yang L, Baddour J, et al. Tumor microenvironment derived exosomes pleiotropically modulate cancer cell metabolism. Elife. 2016;5:e10250. 10.7554/eLife.10250. 26920219 10.7554/eLife.10250 PMC4841778

[CR181] Zhao B, Luo J, Wang Y, et al. Metformin suppresses self-renewal ability and tumorigenicity of osteosarcoma stem cells via reactive oxygen species-mediated apoptosis and autophagy. Oxid Med Cell Longev. 2019;2019:9290728. 10.1155/2019/9290728. 31827709 10.1155/2019/9290728 PMC6885828

[CR182] Zhuang X, Chen Y, Wu Z, et al. Mitochondrial miR-181a-5p promotes glucose metabolism reprogramming in liver cancer by regulating the electron transport chain. Carcinogenesis. 2020;41(7):972–83. 10.1093/carcin/bgz174. 31628462 10.1093/carcin/bgz174

[CR183] Zou H, Chen Q, Zhang A, et al. MPC1 deficiency accelerates lung adenocarcinoma progression through the STAT3 pathway. Cell Death Dis. 2019;10(3):148. 10.1038/s41419-019-1324-8. 30770798 10.1038/s41419-019-1324-8 PMC6377639

[CR184] Zou H, Chen Q, Zhang A, et al. MPC1 deficiency accelerates lung adenocarcinoma progression through the STAT3 pathway. Cell Death Dis. 2019;10(3):148. 10.1038/s41419-019-1324-8. 30770798 10.1038/s41419-019-1324-8 PMC6377639

[CR185] Zy F, Ws W, Sf L, et al. High expression of the TEFM gene predicts poor prognosis in hepatocellular carcinoma. J Gastrointest Oncol. 2020;11(6):1291–304. 10.21037/jgo-20-120. 33457002 10.21037/jgo-20-120 PMC7807266

